# Application of Microfluidic Systems for Breast Cancer Research

**DOI:** 10.3390/mi13020152

**Published:** 2022-01-20

**Authors:** Zachary D. Frankman, Linan Jiang, Joyce A. Schroeder, Yitshak Zohar

**Affiliations:** 1 Department of Biomedical Engineering, University of Arizona, Tucson, AZ 85721, USA; zdfrankman@email.arizona.edu; 2 Department of Aerospace and Mechanical Engineering, University of Arizona, Tucson, AZ 85721, USA; jiangl@email.arizona.edu; 3 Department of Molecular and Cellular Biology, University of Arizona, Tucson, AZ 85721, USA; joyces@email.arizona.edu

**Keywords:** breast cancer, microfluidics, metastasis, dormancy, drug development

## Abstract

Cancer is a disease in which cells in the body grow out of control; breast cancer is the most common cancer in women in the United States. Due to early screening and advancements in therapeutic interventions, deaths from breast cancer have declined over time, although breast cancer remains the second leading cause of cancer death among women. Most deaths are due to metastasis, as cancer cells from the primary tumor in the breast form secondary tumors in remote sites in distant organs. Over many years, the basic biological mechanisms of breast cancer initiation and progression, as well as the subsequent metastatic cascade, have been studied using cell cultures and animal models. These models, although extremely useful for delineating cellular mechanisms, are poor predictors of physiological responses, primarily due to lack of proper microenvironments. In the last decade, microfluidics has emerged as a technology that could lead to a paradigm shift in breast cancer research. With the introduction of the organ-on-a-chip concept, microfluidic-based systems have been developed to reconstitute the dominant functions of several organs. These systems enable the construction of 3D cellular co-cultures mimicking in vivo tissue-level microenvironments, including that of breast cancer. Several reviews have been presented focusing on breast cancer formation, growth and metastasis, including invasion, intravasation, and extravasation. In this review, realizing that breast cancer can recur decades following post-treatment disease-free survival, we expand the discussion to account for microfluidic applications in the important areas of breast cancer detection, dormancy, and therapeutic development. It appears that, in the future, the role of microfluidics will only increase in the effort to eradicate breast cancer.

## 1. Introduction

Cell cultures have been major tools used in cellular and molecular biology for generations, and although extremely useful for delineating cellular mechanisms, are poor predictors of physiological responses [1]. The lack of a proper micro-environment with critical communications between different cell types has been the major reason for this failure [2]. Cells reside in highly specialized extracellular matrices (ECMs) that provide mechanical support, determine mechanical properties, and importantly, impart extracellular signals to the cells. The ECM is the noncellular component present within all tissues and organs, providing not only the essential physical scaffolding for the cellular constituents, but also initiates crucial biochemical and biomechanical cues required for tissue morphogenesis and homeostasis [3]. Cell cultures also suffer from the absence of interstitial flow, which plays an important role in the morphogenesis, function, and pathogenesis of tissues. About 20% of the body’s mass is estimated to be made up of interstitial fluid, and much of this fluid is in constant slow motion. In living tissues, interstitial flow is linked most closely with lymphatic drainage, which returns plasma that has leaked out of the capillaries to the blood circulation [4]. Interstitial flow provides cells with fresh nutrients and removes waste products affecting cell–cell signaling steadily rather than periodically. Animal models, on the other hand, although presenting the same tissue microenvironment, physiology, and molecular interactions of humans, have dissimilar hormone and growth factor milieus, altered drug metabolism, and are difficult to finely tune at the cellular level [5]. These serious shortcomings emphasize the critical need to develop new in vitro biomimetic systems that better represent the in vivo human physiological conditions in effort to hasten biomedical innovation.

Microfluidic technology is emerging as a promising alternative utilized in a wide range of applications in basic and applied biomedical research. Microfluidic devices are useful tools in studying biological phenomena involving fluid flow, thin tissue culture, and cell mobility [6]; each of these aspects make microfluidic techniques attractive for studying human breast cancer, which is the most commonly occurring cancer in women and the second most common cancer overall worldwide [7,8]. Although cancer can arise from any tissue, the ductal epithelium of the breast is of prime interest, because it is the site of 20% of cancers, compared with 15% for the lung epithelium and 13% for the colon epithelium [9]. Identifying and exploiting the distinct properties of tumors is the primary focus of oncology, and microfluidic devices are innately well suited for this goal. In comparison with traditional 2D cell cultures or animal models, microfluidic devices provide smaller scales, greater reproducibility, reduced cost, a lower sample volume, and superior control over the cellular microenvironment [10]. Indeed, microfluidic devices are becoming more prevalent in academia and industry.

Although some cancers may recede into a dormant state of quiescence, many can progress towards metastatic invasion [11]. Metastatic invasion can include local invasion from the primary site, intravasation and extravasation through lymph or blood vessels, and the colonization of a secondary site within the body. Intravasation is the process where tumor cells enter the circulatory system, by invading the endothelial lining of blood or lymph vessels, to become circulating tumor cells (CTCs), enabling them to spread to remote sites in the body [12]. Extravasation is the process by which CTCs exit the circulatory system to settle in a new organ system; breast CTCs have been found to primarily extravasate into four organs, the bone, brain, liver, and lungs, where they then form secondary tumors that may be fatal [13,14]. Breast cancer metastasis is under intense investigation, and a comprehensive review of biomimetic microfluidic platforms developed for its assessment has recently been presented [15]. However, in addition to investigating the mechanisms of metastasis, microfluidic devices are also useful tools in exploring new breast cancer detection and treatment methods, with microfluidic devices having been used to test and troubleshoot various treatment modalities for development of new therapeutic strategies [16,17]. Hence, in this review, we will summarize the applications of microfluidic systems in numerous facets of breast cancer research including metastasis, detection, and treatment.

### 1.1. Breast Cancer Physiology

**Figure 1:**
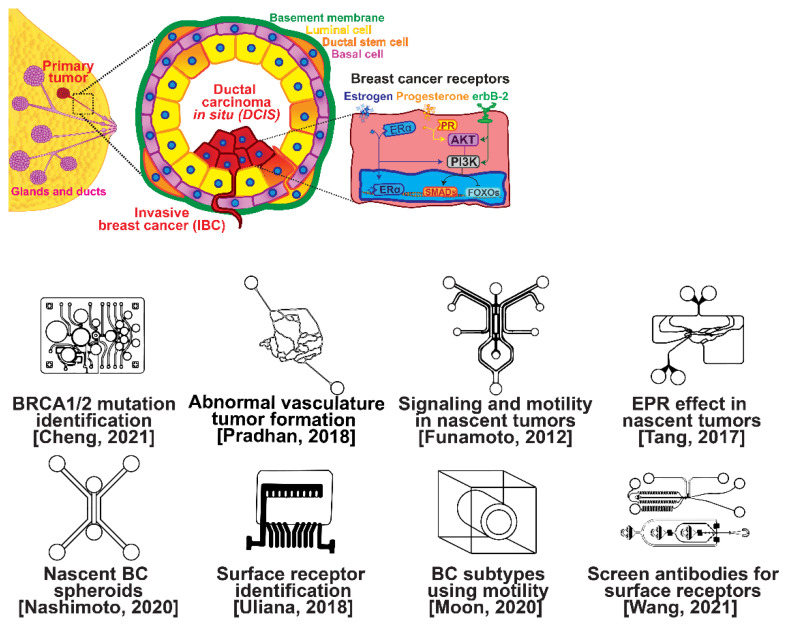
Selected microfluidic platforms for studying breast cancer tumor formation in ducts; normal epithelia lined by a basement membrane can proliferate locally to give rise to a tumor. Further transformation by epigenetic changes and genetic alterations leads to a carcinoma in situ, still outlined by an intact basement membrane. Three canonical surface receptors upregulated in tumor cells have been identified as distinguished biomarkers significant in cancer signaling: the estrogen receptor (ER), the progesterone receptor (PR), and the receptor tyrosine protein kinase ErbB-2, commonly known as HER2.

The ductal epithelium is composed of two epithelial subtypes, the basal or myoepithelium and the luminal epithelium, each of which have distinct markers [18]. The myoepithelium is in direct contact with the basement membrane, which is composed of type IV collagen and proteoglycans (Figure 1, purple cells). The luminal cells are exposed to the ductal lumen (Figure 1, yellow cells), and these cells can undergo hyperplasia and grow into the lumen to form ductal carcinoma in situ (DCIS), a non-invasive and benign, but pre-cancerous condition (Figure 1, red cells). Upon transformation, cells of either the basal or luminal epithelium can invade across the basement membrane and into the surrounding stroma or extracellular matrix, forming an invasive ductal carcinoma. Further alterations in gene expression can then lead to a metastatic cascade, whereupon cells can travel to distant organs and colonize new sites of growth and invasion.

Several microfluidic devices have been developed to study the origin of breast cancer. A microfluidic platform was designed for screening the blood plasma of patients for DNA bearing BRCA1/2 mutations to detect ovarian-cancer-related mutations, as ovarian cancer has a similar mutational origin as breast cancer [19]. Other devices have been developed to investigate early cancer conditions. Breast cancer tumor formation and progression was modeled in a microfluidic system, recreating the pathophysiologic vasculature observed in patients, where low-perfusion physiology was found to enhance tumor progression. The vascular channels, 100 μm in height and width, were separated from the primary and secondary tumor chambers via a 100 μm wide interstitial gap, while the gap was fitted with PDMS pillars of diameter 20 μm and pillar-to-pillar spacing of 20 μm to facilitate cellular interactions [20]. The metabolism of cancer cells during tumorigenesis was explored in a similar device, demonstrating that heterogeneous adaptations to metabolic stress in nascent tumors make anti-cancer drugs targeting cancer metabolism less effective [21]. A microfluidic device was utilized to elucidate the influence of HIF-2 on early cancer motility and signaling [22]. Furthermore, the enhanced permeation and retention (EPR) effect, a controversial concept suggesting that molecules of certain sizes accumulate in tumor much more than they do in normal tissue, has been investigated in breast cancer tumorigenesis using a physiologically relevant vasculature model [23]. Finally, breast cancer spheroids were formed in a generalized breast tumor analysis microfluidic platform for early tumor physiological research and drug discovery [24].

### 1.2. Breast Cancer Types

Types of breast cancer include ductal carcinoma in situ (DCIS), invasive ductal carcinoma (IDC), inflammatory breast cancer (IBC), and metastatic breast cancer (MBC). Once established, breast cancer cells can be distinguished from their surrounding tissue by their distinct surface receptor expression. Three canonical surface receptors have been identified as being critical biomarkers and significant in cancer signaling—the estrogen receptor (ER), the progesterone receptor (PR), and the receptor tyrosine protein kinase ErbB-2, commonly known as HER2. These are excellent biomarkers because of the contrast in their expression within invasive breast cancer compared with healthy tissue—ER is upregulated in ~75% of IBCs, with PR upregulated in ~70% and HER2 upregulated in 10–40% [25]. However, ~10% of breast cancers do not exhibit upregulation of the ER, PR, or HER2 receptors; therefore, they are designated as triple-negative breast cancers (TNBCs), which receive signaling through a combination of less common pathways [26,27]. The ER, PR, and HER2 receptors related to breast cancers are significant in that they are highly expressed in contrast to surrounding tissue and engage with well-studied signal cascades. This enables reliable prediction of the cancer behavior, and the use of pharmacological therapy to halt the cancer from aberrant signaling expression [28,29,30,31]. Receptor expression disparity is the basis of several microfluidic platforms developed for breast cancer research, including an inexpensive and disposable microfluidic system that was constructed to enable the probing of cancer cells based on these surface receptors. The fully disposable microfluidic electrochemical array device was constructed using low-cost materials, and an inexpensive home cutter printer enabled the manufacture of dozens of devices in less than 2 h, at a cost of less than USD 0.20 in material per device [32].

## 2. Breast Cancer Metastasis

Metastasis is one of the hallmarks of cancer, distinguishing it from benign tumors [33]; the term is used to describe the spread of cancer from its original/primary site to a different/secondary site within the body. Unlike normal cells, cancer cells possess the ability to grow outside of the tissue in the body where they originated. Nearly all cancer types can metastasize, but whether they do metastasize depends on many factors. Metastases can occur in three ways: (i) grow directly into the tissue surrounding the tumor, (ii) travel through the lymph system to nearby or distant lymph nodes, or (iii) travel through the blood stream to distant locations. Metastasis, in general, involves invasion, intravasation, and extravasation, which will be discussed together with the application of microfluidic technology to research in each of these phases.

### 2.1. Invasion Modeling

**Figure 2:**
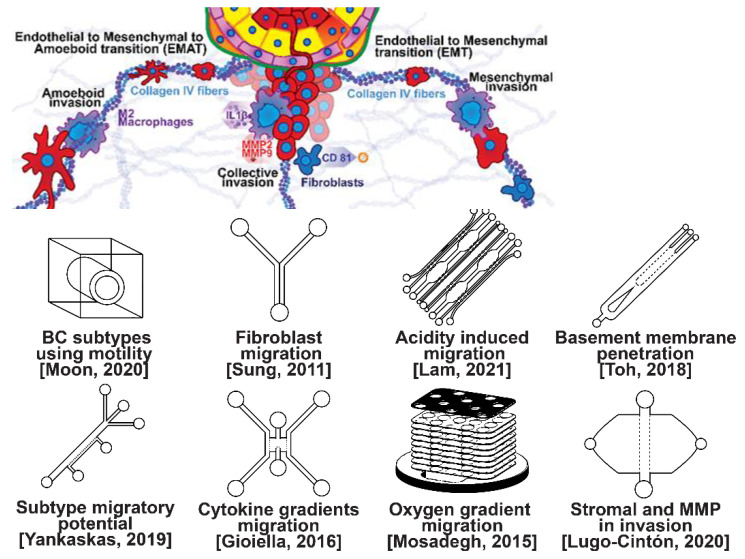
Selected microfluidic platforms for studying breast cancer invasion, the first stage of metastasis, which occurs as cancer cells break off from the primary breast tumor to invade the surrounding tissue. As proliferation increases, cancer cells become confined, hypoxic, and metabolically starved. These factors contribute to an epithelial-to-mesenchymal (EMT) transition within the cancer cells, changing their morphology and greatly increasing their motility. Aside from the primary stimuli of invasion: (i) highly motile amoeboid, (ii) slow collective, and (iii) mesenchymal cell migration, cancer cells have also been shown to become invasive or undergo EMT in response to acidity, stromal and endothelial cell crosstalk, healthy immune cell signaling, excess ECM density, and some forms of chemo- or radiotherapy.

Invasion occurs as cancer cells begin to break off from the bulk of the tumor to invade its surrounding tissue as illustrated in Figure 2. As proliferation increases, cancer cells become confined, hypoxic, and metabolically starved [34,35]. These factors contribute to an epithelial-to-mesenchymal (EMT) transition within the cancer cells, changing their morphology and greatly increasing their motility [36]. Recently, E-cadherin was singled out as required for invasion in multiple models of breast cancer, which could still involve partial/hybrid EMT states [37]. Confinement has also been found to induce cancer cells to enter a highly motile amoeboid state that initiates the invasion of surrounding tissue, but invasion may also occur through slower collective and mesenchymal cell migration [38,39]. Aside from these primary stimuli, cancer cells have also been shown to become invasive or undergo EMT in response to acidity, stromal and endothelial cell crosstalk, and excess ECM density [40,41,42]. Invading breast cancer cells begin to degrade restrictive extracellular matrix components by expressing matrix metalloproteinases, such as MMP-2 and MMP-9, and similar matrix reconstruction enzymes that lead to widespread breast cancer invasion [43].

Microfluidic devices allow for improved characterization of the factors related to breast cancer invasion. The migratory potential of cancer subtypes was examined in a microfluidic device based on their distinct surface receptor combinations [44]. Microfluidics has also been used to identify alternative surface receptors for further investigation, and specific subtypes were identified by observing their migratory patterns [45]. A microfluidic 3D compartmentalized system was introduced to co-culture mammary epithelial cells (MCF-DCISs) with human mammary fibroblasts (HMFs), promoting a transition from DCIS to IDC in vitro [46]. The model enabled the control of both spatial and temporal features of the microenvironment, thereby, recapitulating the in vivo environment in ways not practical with existing experimental models. Analysis of intrinsic second harmonic generation signal of collagen allowed the label-free quantitative analysis of DCIS-associated collagen remodeling. Moreover, the arrayed microchannel-based model provided a cost-effective test bed for identifying inhibitors of pathways involved in DCIS progression to IDC and for screening potential therapeutic targets. Calcium carbonate nanoparticles were found to stimulate cancer cell reprogramming to suppress tumor growth and invasion in an organ-on-a-chip system, which was developed to create a tumor microenvironment for isolating the effect of pH on tumor viability [47]. The results suggested that treating breast cancer cells (MDA-MB-231) co-cultured with fibroblasts using CaCO_3_ nanoparticles can restrict the aggressiveness of tumor cells without affecting the growth and behavior of the surrounding stromal cells. Breast cancer cells were observed to penetrate the duct basement membrane at the origin of invasion in a 3D microfluidic model designed to recapitulate cancer cell migration and invasion [48]. Similarly, using a microfluidic assay for the quantification of the metastatic propensity of breast cancer specimens, high invasive potential was found to be correlated with the RAS/MAPK and PI3K pathways, as previously suggested [49]. Additionally, the in vivo cytokine gradients were recreated in a microfluidic tissue culture model that permitted the visualization of various breast cancer subtypes infiltrating the surrounding tissue [50], and cancer cells were observed to preferentially invade towards regions of higher oxygen concentrations in a paper-based microfluidic device [51]. Utilizing microfluidic devices, tumor-associated macrophages (TAMs) and U-937 cells were found to promote tumor invasion [52], and breast cancer stromal cells were reported to interact with migratory tumor cells to facilitate their motility by secreting MMPs at levels that overpower anti-MMP drugs [53]. Qualitative and quantitative mapping of a large population of cell–protein interactions were carried out in a microfluidic platform; the upregulated β1 integrin in invasive cancer cells enhanced cell–ECM interaction, promoting its remodeling, and cancer cells showed strong interaction with plasma fibrinogen that may support their arrest on blood vessels [54].

### 2.2. Intravasation Modeling

**Figure 3:**
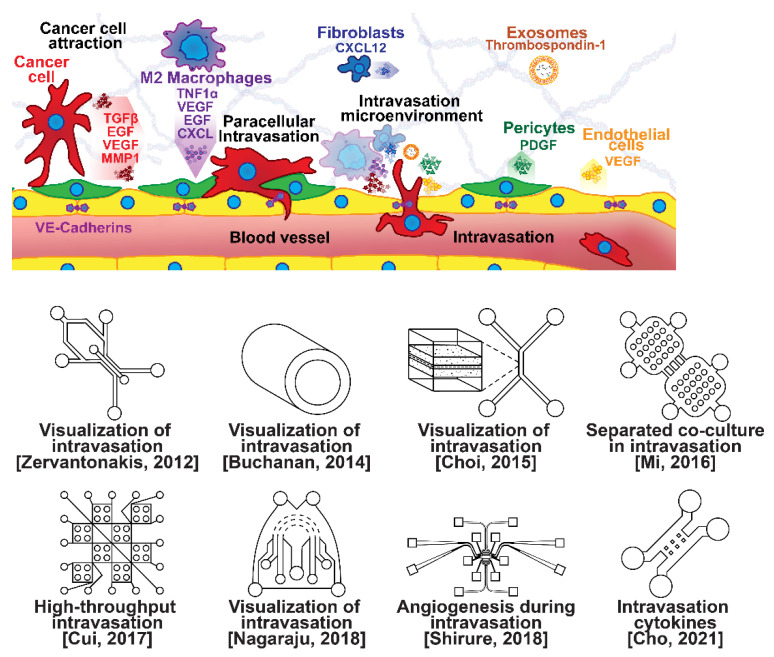
Selected microfluidic platforms for studying breast cancer intravasation, the process by which invading cancer cells enter a blood or lymphatic vessel allowing their passive transport to distant organs as CTCs. Once capable of moving through the ECM, motile breast cancer cells have been found to follow collagen fibers that lead from their primary site to nearby blood or lymph vessels. However, breast cancer cells are often incapable of penetrating the basal lamina or the endothelial cell layer surrounding the lumen of these vessels due to cadherins forming tight intercellular junctions. This seems to be overcome with the assistance of various signaling pathways and macrophage interactions forming a microenvironment that enables cancer cells to penetrate the vasculature.

Once able of moving through the ECM, motile breast cells have been found to follow collagen fibers that lead from their primary site to nearby blood or lymph vessels [55]. However, breast cancer cells are often incapable of penetrating the basal lamina or the endothelial cell layer surrounding the lumen of these vessels due to cadherins forming tight intercellular junctions [56,57]. This seems to be overcome with the assistance of various signaling pathways and macrophage interactions forming a microenvironment that enables cancer cells to penetrate into the bloodstream, as shown in Figure 3 [56,57,58,59,60]. Macrophages in the tumor environment display a wide phenotypic spectrum, varying from anti-tumor M1 types to pro-invasion M2 types [61]. Pro-invasion M2-like tumors express factors that lower cadherin density in vascular endothelial cells, such as the angiogenic signals TNF1α, VEGF, and EGF, as well as immune-cell recruiting signals such as the CXCL family, all of which prime the vessel for penetration [60,61,62,63,64]. Tumor cells themselves actively participate in the intravasation process through a variety of means, e.g., via the NOTCH and TGFβ1 pathways, to induce cadherin degradation and endothelial contraction, permitting extravasation [65,66,67]. Breast cancer cells secreting micro-RNA (miRNA) signals, such as miR-939, have been under investigation due to their role in stimulating endothelial permeability [68,69].

Microfluidic devices are attractive for investigating intravasation in breast cancer because they allow precise spatial control of cell positioning. Using an engineered microfluidic migration chamber, MDA-MB-231 breast cancer cell invasion through confined microchannels was shown to induce a change in migratory phenotype [70]. To recreate the tumor–vascular interface in three dimensions, enabling precise quantification of the endothelial barrier function, a microfluidic-based assay was developed for studying the regulation of carcinoma cell intravasation by biochemical factors from the interacting cells and cellular interactions with macrophages; endothelial permeability measurements showed that signaling with macrophages via the secretion of tumor necrosis factor alpha results in endothelial barrier impairment [71]. In another co-culture study, employing a 3D cylindrical configuration coupled with confocal microscopy, tumor cells were found to significantly increase the expression of proangiogenic genes in response to co-culture with endothelial cells under low flow conditions. Using microparticle image velocimetry (μ-PIV), the flow rate was adjusted to be in the range of 260–2600 μL/min to generate a target wall shear stress of 1–10 dyn/cm^2^ [72]. Such a system provides a downstream molecular analysis capability which can serve as a versatile platform for elucidating the role of fluid forces on tumor–endothelial crosstalk. A biomimetic microengineering strategy was described to reconstitute 3D structural organization and the microenvironment of breast tumors in human-cell-based in vitro models. The microsystem enabled the co-culture of breast tumor spheroids with human mammary ductal epithelial cells on one side of an ECM membrane, and mammary fibroblasts on the other side of the ECM membrane, in a compartmentalized microfluidic device to replicate the microarchitecture of breast ductal carcinoma in situ (DCIS) [73]. A microfluidic co-culture system was presented for the establishment of mild, moderate, and severe cancer models to study cancer cell migration. The density of the cancer cells was reported to determine the probability of the occurrence of metastatic cells as well as their velocity, with the increase in the migration velocity of MDA-MB-231 cells co-cultured with HMEpiC cells found to be proportional to the increased secretion of IL-6 [74]. Trans-endothelial migration has also been explored using a high-throughput multi-channel microfluidic device [75]. A 3D microfluidic platform comprising concentric three-layer cell-laden hydrogels was developed for the simultaneous investigation of breast cancer cell invasion and intravasation as well as vasculature maturation influenced by tumor–vascular crosstalk. It was demonstrated that the presence of a spontaneously formed vasculature enhanced MDA-MB-231 invasion into the 3D stroma. The invading cancer cells significantly reduced vessel diameter while increasing permeability, and major signaling cytokines involved in tumor–vascular crosstalk governing cancer cell invasion and intravasation were identified [76]. To dynamically observe tumor progression, including cell migration, angiogenesis, and tumor cell intravasation, a microfluidic platform was realized for mimicking biological mass transport near the arterial end of a capillary in the tumor microenvironment [77]. However, most in vitro metastasis models favor investigating blood-vessel-based metastasis pathways; thus, the understanding of lymphatic metastasis is limited, which is also closely related to the inflammatory system. To understand the effects of inflammatory cytokines in lymphatic metastasis, a three-channel microfluidic system was constructed to mimic the lymph vessel–tissue–blood vessel (LTB) structure; each channel was about 100 μm in width. Matrigel was injected into the middle channel to mimic the tissue, while human umbilical vein endothelial and human lymphatic endothelial cell layers were formed in the side channels to reconstitute blood and lymph vessels, respectively. The exposure of different subtypes of breast cancer cells to an inflammatory cytokine, interleukin 6 (IL-6), induced epithelial–mesenchymal transition and enhanced tissue invasion. Similar LTB chips could be applied to analyze the intercellular communication in the tumor microenvironment under various extracellular stimuli such as inflammatory cytokines, stromal reactions, hypoxia, and nutrient deficiency [78].

### 2.3. Extravasation Modeling

**Figure 4:**
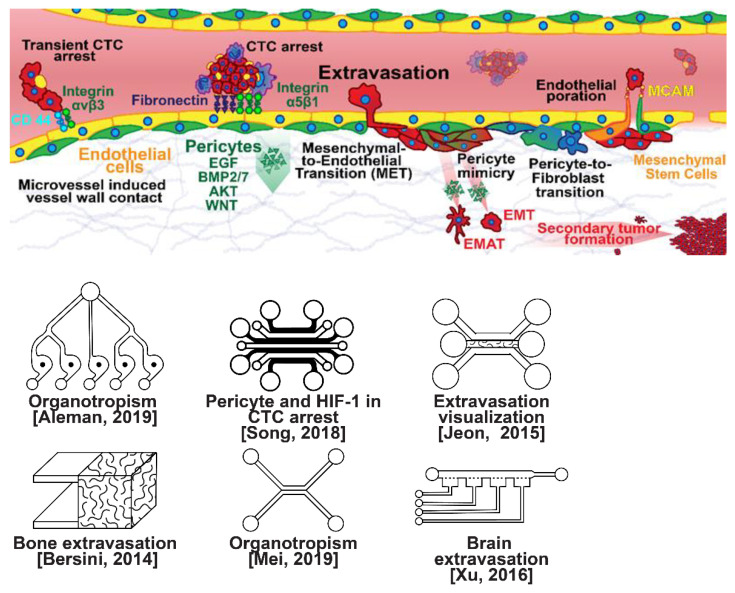
Selected microfluidic platforms for studying breast cancer extravasation, the process by which CTCs exit the vasculature in remote host organs. Individual CTCs resist immune system attacks through a myriad of mechanisms, such as the degradation of apoptotic receptors and the expression of attack-arresting surface markers. However, individual CTCs may undergo apoptosis under the influence of immune cytokines and fluid shear forces, and undergo anoikis upon loss of attachment to the extracellular matrix (ECM) and neighboring cells due to a lack of fibronectin mediation. CTCs that aggregate have been shown to be more resilient, particularly when the aggregates include platelets and neutrophils which disguise them. At remote sites, solitary carcinoma cells can extravasate through either endothelial poration or pericyte-mediated recruitment; they then may remain solitary (micrometastasis) or form a new secondary tumor through EMT (macrometastasis).

**Figure 5:**
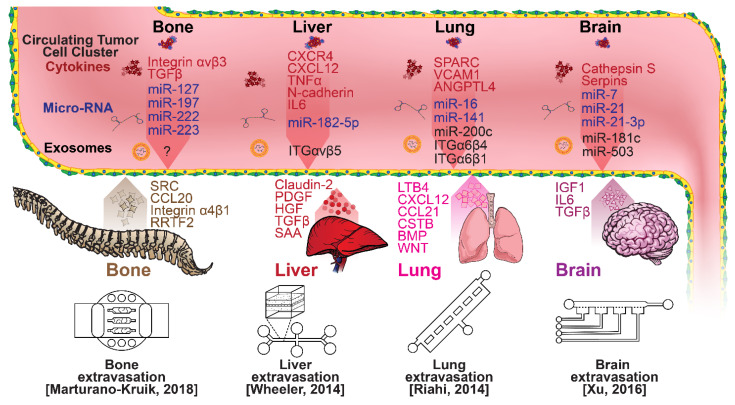
Selected microfluidic platforms for studying breast cancer organotropism, which is the organ-specific extravasation of CTCs involving: (i) the release of pro-inflammatory cytokines into circulation by the primary tumor, (ii) the upregulation of endothelial adhesion molecules at secondary sites induced by the cytokines, (iii) CTCs adhering to and migrating through endothelial cells and proliferating. Breast CTCs have been found to primarily extravasate into the bone, liver, lungs, and brain, where they form secondary tumors that may be fatal.

Once within a lymph or blood vessel, tumor cells become circulating tumor cells (CTCs) that are targeted by different immune cells to varying extents [79]. Individual CTCs resist immune system attacks through a myriad of mechanisms, such as the degradation of apoptotic receptors such as TRAIL, and the expression of attack-arresting surface markers such as CD47 and PD-L1 [80,81,82]. However, individual CTCs may undergo apoptosis under the influence of immune cytokines and fluid shear forces, and undergo anoikis upon loss of attachment to the extracellular matrix (ECM) and neighboring cells due to a lack of fibronectin mediation [83,84]. CTCs that aggregate through CD-44 cohesion have been shown to be more resilient, particularly when the aggregates include platelets and neutrophils which disguise them [85,86]. Heterogenous cell aggregates also lead to CTC proliferation through IL-1β and IL-6 crosstalk [87]. CTCs spread with circulation to remote sites and, subsequently, they can extravasate from the vascular system through blood vessel walls into the surrounding tissue forming tumors at host organs, as shown in Figure 4. The site of CTC arrest is known as the secondary site and is found to occur primarily in selective organs. This targeted arrest is known as organotropism and is described in Figure 5, with breast cancer secondary sites appearing mainly in the bone, brain, liver, and lungs, in addition to the axillary lymph nodes, which are diagnostic for metastatic disease in ~97% of patients [13,14,88]. Organotropism is presumed to originate from the primary tumor secreting context-dependent signals to distal sites that sensitize them to receive CTCs which, in cooperation with neutrophils, seek the pre-conditioned sites [89,90,91]. Certain signals have been implicated with specific organotrophic targets, such as TGFβ and COX2 for the lungs, SRC-dependent pathways for bone, and IGF1 for the brain [92,93,94]. mi-RNAs have also been identified as significant in targeted organotropism, with miR-16/148a for the lungs and miR-127/197/222/223 for bone [89,90,91]. Tumor exosomes may be involved in organotropism; tumor-derived exosome uptake by organ-specific cells was observed to prepare the pre-metastatic niche, and treatment with exosomes from lung-tropic models redirected the metastasis of bone-tropic tumor cells. Moreover, exosome proteomics revealed distinct integrin expression patterns, in which the exosomal integrins α6β4 and α6β1 were associated with lung metastasis, whereas exosomal integrin αvβ5 was linked to liver metastasis. Thus, targeting integrins α6β4 and αvβ5 decreased exosome uptake as well as lung and liver metastasis, respectively. Exosome integrin uptake by resident cells was found to activate Src phosphorylation and pro-inflammatory *S100* gene expression [95]. Outside of chaperoned extravasation, CTCs and CTC clusters may arrest along the vasculature through weak CD-44 and integrin αvβ3 binding, and are then reinforced with stronger fibronectin and integrin α5β1 bonds over time [96]. Once the CTCs affix to their target organ, a similar process to intravasation then occurs, where CTCs excrete signals with neutrophils to permeabilize endothelial cells for infiltration [97]. Trans-endothelial migration enables cancer cells to embed between endothelial cells, mesenchymal stem cells (MSCs), and vascular pericytes [98]. This perivascular niche produces supportive signals such as PI3K, and is a main focus of anti-cancer research [99,100]. Pericytes also play a significant role in breast cancer extravasation, and have been shown to respond to tumor-derived signals by performing an embryogenesis-derived program of angiotrophic migration and fibroblast differentiation, enhancing tumor extravasation and promoting pericyte mimicry (PM) in cancer cells [101,102,103]. Pericyte- and MSC-expressed MCAM/CD146 were reported to alter the expression of the estrogen receptors ErbB3 and ErbB4, resulting in an increase in chemoresistance, EMT, and a worse prognosis, in contrast to previous findings [104,105,106,107]. Confinement within the perivascular niche has been found to induce cancer cells to enter a motile amoeboid morphology that initiates the invasion of surrounding tissue, although invasion may also occur through slower collective and mesenchymal cell migration modes [38,39]. Signals such as EGF, BMP2/7, AKT, and WNT may then reverse EMT by producing a mesenchymal-to-epithelial transition (MET) or amoeboid-to-epithelial transition (AET), indicated by a FGFR2b/FGFR2 switch that leads to migratory arrest and tumorigenesis [108,109].

Microfluidic devices could provide a powerful tool for improving the scientific understanding of CTC behavior, organotropism, and extravasation. A microfluidic method was introduced for the integrated capture, isolation, and analysis of membrane markers, as well as the quantification of proteins secreted by single CTCs and CTC clusters. The proposed platform was tested with multiple breast cancer cell lines spiked into human blood and mouse-model-derived CTCs. The quantified secretion level of granulocyte growth-stimulating factor (G-CSF), which is involved in neutrophil recruitment, was found to be highly expressed across cancer cell lines. Incorporating barcoded magnetic beads, this platform can be adapted for multiplexed analysis enabling comprehensive functional CTC profiling [110]. A thorough investigation of CTC–neutrophil adhesion was conducted using droplet formation techniques, which showed that CTC–neutrophil aggregates upregulated the expression of VCAM-1, E-cadherin, and macrophage recruitment cytokines such as CCL4/24/22 and PPBP [111]. Utilizing a commercially available hepatic microphysiologic system (LiverChip, CN Bio Innovations Limited, Oxford, UK), the liver system was established as a relevant microfluidic model for the study of breast cancer metastasis [112]. A microfluidic system was developed and characterized for the in vitro systematic studies of organ-specific extravasation of CTCs. The system recapitulated the two major aspects of the in vivo extravasation microenvironment: local signaling chemokine gradients in a vessel lined up with an endothelial monolayer. The system was utilized to demonstrate the extravasation of CXCR4-expressing MDA-MB-231 cancer cells, across a confluent HUVEC monolayer, in the presence of CXCL12 chemokine gradients. Consistent with the hypothesis of organ-specific extravasation, control experiments were presented to substantiate the observation that the MDA-MB-231 cell migration was due to controlled chemotaxis rather than a random process [113]. A multi-site metastasis-on-a-chip microphysiological system was described for assessing the metastatic preference of cancer cells. The device housed multiple bioengineered 3D organoids established by a 3D photopatterning technique employing extracellular-matrix-derived hydrogel biomaterials. Under recirculating fluid flow, tumor cells grew in the primary site, entered circulation, and preferentially homed to specific organ constructs. The platform can be implemented to better understand the mechanisms underlying metastasis and, perhaps, leading to the identification of targets for intervention [114]. The impact of hypoxia, a common feature of the tumor microenvironment, on the extravasation potential of breast cell lines, was studied in a 3D microvascular network model. Using HIF-1α knock-down cell lines, the importance of HIF-1α in the transmigration ability of human breast cell lines was validated. Under hypoxic conditions, the HIF-1α protein level was increased, and coincided with changes in cell morphology, viability, and an elevated metastatic potential; these changes were accompanied with an increase in the rate of extravasation compared with normoxia (21% O_2_). Such a microfluidic model can be a reliable in vitro tool for systematically interrogating individual factors and their accompanying downstream effects, which may otherwise be difficult to study in complex tumor tissues [115]. A physiologically relevant vascularized bone matrix to model CTC extravasation into the perivascular niche was created in a similar device, which was then used to demonstrate the anti-metastatic role of interstitial flow [116]. Other projects on the perivascular niche added to the investigation of extravasation by providing alternative designs for imaging and quantifying CTC extravasation [116,117,118,119]. A dynamic in vivo-like 3D microfluidic system replicating key structural, functional, and mechanical properties of the blood–brain barrier (BBB) in vivo was constructed to probe metastatic brain tumors. Multiple factors in this organotypic BBB model work synergistically to accentuate BBB-specific attributes with the complex microenvironment reproduced via physical cell–cell interaction and vascular mechanical cues. The interactions between cancer cells and astrocytes in the BBB microenvironment seemed to affect the ability of malignant brain tumors to traverse between brain and vascular compartments. The model offers the capability of examining brain metastasis of human breast cancer cells and their therapeutic responses to chemotherapy. Furthermore, the quantification of spatially resolved barrier functions exists within a single assay, providing a versatile platform for pharmaceutical development, drug testing, and neuroscientific research [120].

## 3. Detection Techniques of Breast Cancer

Breast cancer is potentially deadly, but has a more positive prognosis if detected and treated early into the metastatic cascade [121,122]. Several techniques are currently available for breast cancer diagnosis, including non-invasive imaging: (i) sonography using sound waves, (ii) mammography using X-rays, and (iii) MRI using magnetic resonance for the detection of breast abnormalities, and invasive biopsy requiring the removal of tissue or fluid from the breast for microscopic testing. In nuclear medicine, using special cameras to inspect the path of injected radioactive substance, the scans depend on tissue-metabolism rather than structural changes due to tumors. Therefore, unlike other imaging modalities, this method provides information on the physiological function of the system. The common techniques are positron emission tomography (PET), single-photon emission computed tomography (SPECT), and scintimammography. Other scanning methods include electrical impedance scanning and thermal imaging. The issues and challenges associated with these techniques, as well as some emerging experimental tests, have been reviewed [123]. The best way to improve the prognosis of breast cancer is early detection; therefore, convenient and inexpensive detection through liquid biopsy has attracted major research and commercial interest to facilitate frequent screenings for breast cancer [124]. Although liquid biopsy is less frequently used than other techniques, it does offer many promising benefits, including reduced cost, greater convenience, and greater sensitivity [125]. The need for low-abundant blood and serum cancer diagnosis with microfluidic tools and the progress in developing integrated microfluidic platforms to meet this need has recently been summarized [126].

### 3.1. Detection of Breast Cancer CTCs

CTCs are found in blood as independent cells, CTC clusters, and diverse clusters composed of neutrophils, platelets, and CTCs [127]. Isolated CTCs are rapidly identified and destroyed by natural killer (NK) cells or phagocytized by macrophages [79]. However, isolated CTCs are formed at twice the rate of CTC clusters, and therefore are the primary target of liquid biopsy studies [97,128]. CTCs experience three major obstacles while suspended in blood, namely, anoikis, immune attack, and hydrodynamic shear stress [12]. CTCs may express certain cell surface receptors (RTKs), such as IGF1R and TRKB, which sensitizes them for the stimulation of survival-inducing pathways to counteract the loss of FAK signaling from anoikis [129,130]. CTCs may also express VCAM-1, which attaches to monocytes, and MICA/B, which promotes apoptosis in attacking NK neutrophils [131,132,133]. CTCs have been found to express LDH5 to communicate with nearby monocytes, stimulating the induction of NKG2D ligands to disrupt NK neutrophil activation [134]. The anchoring of monocyte α4 integrins through CTC-expressed VCAM-1 engages Ezrin/PI-3K signaling to promote survival [132]. Similarly, CTCs were reported to express proteins recruiting platelets, such as TF, ADAM9, and PDPN, which were proposed to form a physical shield around tumor cells protecting them from shear stress and cytotoxic effects of NK neutrophils [135,136,137]. CTCs can withstand fluid shear forces by expressing Pannexin-1 to stimulate autocrine P2Y signaling [138], and can aggregate to each other to form clusters through homophilic CD-44 anchoring to stimulate PAK2/FAK signaling [86], both promoting survival. Cytokine biomarkers of breast CTCs used in clinical detection include easily accessible membrane-localized biomarkers such as EpCAM/CD326 for epithelial CTCs in early metastasis, RGD-peptide-enriched vimentin and fibronectin for mesenchymal CTCs in advanced metastasis, CK-8/18/19 as general non-native blood cell marker, and CA15-3 as a breast-cancer-specific surface marker [139,140,141,142]. Blood cells negative for the leukocyte common antigen CD45 also indicate cancer origin [143].

In terms of physical properties, breast cancer CTCs have a diameter on the order of 7–9 µm and a cross-sectional area of 40–65 µm^2^, putting them at a scale similar to white blood cells (5 µm/20 µm^2^ for small leukocytes and 20 µm/315 µm^2^ for monocytes) [144]. A more recent investigation determined that, in breast cancer, the median computed diameter (CD) of patient-derived CTCs was 12.4 μm vs. 18.4 μm in cell line cultures, whereas leukocytes were 9.4 μm in diameter [145]. Breast cancer CTC clusters from human blood samples are much larger, and are frequently composed of 1–20 CTCs, 40% of which contain attendant platelets, neutrophils, and monocytes to reach aggregations at an average of 80–350 µm in diameter, permitting size exclusion for isolation [128,146]. Small clusters (2–5 cells) are likely to be elongated and asymmetrical due to the pseudospherical shape of cells and the improbability of symmetrical attachment, which alters flow behavior and permits isolation using vorticity [147]. CTCs are distinguished by their low mass density, lower than 1.08 g/mL, in comparison with denser white and red blood cells allowing enrichment in plasma through centrifugation [148]. The Young’s modulus values of CTCs are reportedly in the range of 6.2–23.0 kPa, with lower stiffness associated with higher metastatic potential [149]. This is substantially stiffer than the 0.00004–0.02 kPa of leukocytes, which can be used for deformability exclusion [150,151]. Another distinguishing physical biomarker of CTCs is their unique ion channel composition in comparison with native blood cells, conferring them with distinct electrical properties. Isolated metastatic human breast cancer cells have a conductance of 0.34 S/m and a capacitance of 14.83 mF/m^2^, compared with 0.31–0.53 S/m conductivity and 7.01–11.77 mF/m^2^ capacitance of leukocytes, enabling dielectric exclusion techniques [152].

**Figure 6:**
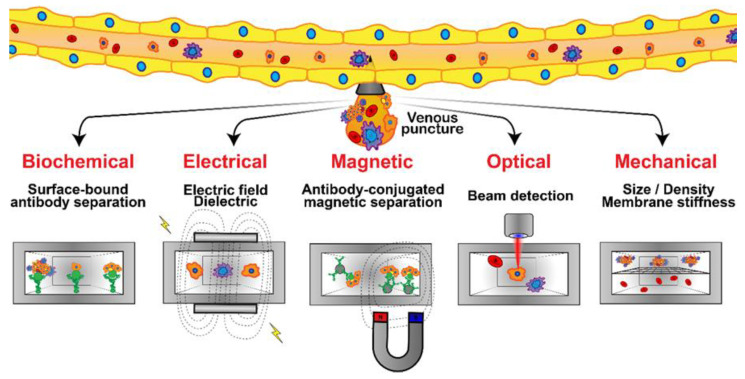
Microfluidic platforms developed for CTC detection and isolation from whole blood samples are based on several techniques: bio-chemical (specific receptor–ligand interaction), mechanical, electrical, optical, and magnetic. Immunostaining assays or genetic analyses are then applied to identify, enumerate, and characterize the isolated CTCs.

The clinical significance of CTCs in metastatic breast cancer has been clearly established [153]. CTCs are rare, comprising as few as one cell per 10^9^ hematologic cells in the blood of patients with metastatic cancer, which is equivalent to fewer than 100 cells per ml of blood. Hence, their isolation presents a tremendous technical challenge [154]. Although extremely rare, CTCs represent a potential alternative to invasive biopsies as a source of tumor tissue for the detection, characterization and monitoring of non-hematologic cancers [155]. Several technologies have been proposed for cell separation from whole blood in the last decade, and microfluidic technology has been extensively utilized to exploit each chemical or physical biomarker for isolating CTCs (Figure 6) [156]. Microfluidic devices exploiting size, asymmetry, and elasticity differentials to enrich and isolate CTCs, with a narrow gap as small as 5 µm, as well as CTC aggregates from whole blood, with openings as wide as 12 µm, have been demonstrated [147,157,158]. Furthermore, CTC clusters were reported to break apart under shear forces in microfluidic devices, reducing metastatic potential [159,160]. A microfluidic-elasto-filtration device based on the critical elasto-capillary number has been proposed to utilize the optimal multi-parameter conditions, including cell diameter, the diameter of cylindrical filter pores, nonlinear cell elasticity and hydrodynamic drag force, for selectively capturing CTCs and depleting white blood cells (WBCs) [161]. Dielectrophoresis (DEP) is emerging as an alternative method for isolating CTCs from blood. This electrokinetic method allows the intrinsic dielectric properties of suspended cells to be exploited for discrimination and separation. DEP-isolation of CTCs is independent of cell surface markers, and the isolated CTCs are viable and can be maintained in culture [162]. Low-cost and rapid-production microfluidic electrochemical double-layer capacitors have enabled fast and sensitive breast cancer diagnosis using the dielectric properties of CTCs [163]. Magnetic sorting using magnetic beads has become a routine methodology for the separation of key cell populations from biological suspensions. Due to the inherent ability of magnets to provide forces at a distance, magnetic cell manipulation is now a standardized process step in numerous processes in tissue engineering, medicine, and in fundamental biological research. The status of magnetic particles to enable the isolation and separation of cells, with a strong focus on the fundamental governing physical phenomena, properties, and syntheses of magnetic particles and on the current applications of magnet-based cell separation in laboratory and clinical settings, has been reviewed [164]. Applications of mechanical, electric, magnetic, and acoustics-based methods for sorting and manipulation of cells within the framework of a microfluidic devices are under continuous development, and have been reviewed in numerous articles [165]. Optics-based methods, in contrast, have not been explored to the same extent as other methods. They attracted insufficient attention due to the complicated, expensive, and bulky setup required for carrying out such experiments. However, some advances in contemporary techniques using light for cell sorting and manipulation have been outlined [166]. To avoid foreign material from causing damage while maintaining high efficiency, an innovative strategy has been proposed using tumor-cell-targeting molecules to bind homologous red blood cells (RBCs) with tumor cells. This has resulted in optical constant differences (both size and mean refractive index) between the RBC-conjugated CTCs and other blood cells. The modified CTCs were subsequently separated, under laser illumination in an optofluidic system, at high purity and recovery rates without compromising their membrane and function integrity [167].

Biochemical interactions have been the basis for the most widely researched CTC isolation techniques in microfluidic devices, relying on specific cell receptor–ligand binding and, often, antibodies have been employed as the counter-receptor ligands [155]. In the affinity-based processing of homogeneous CTC suspensions under flow in microchannels, the fraction of captured cells was reported to mainly depend on the flow shear rate with a characteristic shear rate depending on both cell–receptor and surface–ligand densities [168]. Furthermore, to enhance the efficiency of CTC isolation from heterogeneous suspensions, a two-step attachment/detachment flow field pattern was implemented, combining a slow flow field, for maximum target-cell attachment, followed by a faster flow field, for the maximum detachment of non-target cells [169]. The prognostic relevance of CTCs in metastatic breast cancer has been demonstrated using a flow-based microfluidic separation platform. CTC count before treatment was proposed as an independent predictor of progression-free survival in patients with HER2-negative MBC, supporting the clinical validity of CTC detection using the label-free microfluidic platform [170]. Microfluidics has proven to be a leading technology for the capture of CTCs and their downstream analysis, with the aim of shedding light on their clinical application in cancer metastasis. The key aspects of affinity and label-free microfluidic CTC technologies have been scrutinized in detail, suggesting that integrated techniques would be more beneficial [171].

### 3.2. Detection of Breast Cancer Biomarkers

In addition to breakaway tumor cells, breast cancer may also be identified through subcellular signals such as exosomes, DNA, RNA, and proteins [172]. Exosomes are nanometer-scale vesicles expressing surface markers such as integrins, tetraspanins, and immunoregulatory markers [173,174,175]. Breast-cancer-associated exosomes are often found to contain RNA (nc-RNA, mi-RNA, and m-RNA), DNA, signaling molecules such as B-catenin or EGF, enzymes such as GAPDH, and chaperones such as HSP60/70/90 or Cyclophilin A [176,177,178,179,180,181,182]. Exosomes influence virtually every aspect of cancer development, including immunosuppression, proliferation, and drug resistance [183,184,185]. Free-floating RNA and methylated DNA signals produced by cancer cells are also linked to cancer function and, sometimes, are used as prognostic biomarkers [172,186].

Exosomes are promising biomarkers for liquid biopsies, because these nano-sized extracellular vesicles enrich proteins, lipids, mRNAs, and miRNAs from cells of origin, including cancer cells. Although exosomes are abundantly present in various bodily fluids, conventional exosome isolation and detection methods that rely on benchtop equipment are time-consuming, expensive, and involve complicated non-portable procedures. Therefore, microfluidic devices designed for exosome and serum signal detection are of major interest in oncology, and a diverse array of isolation techniques including immunoaffinity, acoustic, size exclusion filtration, and electrokinetic capture have recently been explored [187]. For the specific detection of exosomes in blood, a multiplexed device based on electrohydrodynamic nanoshearing has been developed to greatly reduce nonspecific binding [188]. For the real-time and label-free profiling of clinically relevant exosomes, surface plasmon resonance was utilized in a microfluidic chip to quantify exosomes and enable proportional measurements of surface biomarker expression by immunofluorescence [189]. Clinical applications of a microfluidic chip have been proposed for the immunocapture and quantification of circulating exosomes to assist breast cancer diagnosis and molecular classification [190]. Efficient isolation and quantification of extracellular vesicles has been accomplished in an integrated ExoID-Chip using ultrasensitive photonic crystals [191], and a microfluidic chip has been applied for the multiple detection of miRNA biomarkers in breast cancer based on three-segment hybridization using fluorescent imaging [192]. These integrated platforms offer advantages of integrity, speed, cost-efficiency, and portability over conventional methods, which can only separate or detect exosomes separately. Hence, with further development for liquid biopsies, such microfluidic devices could be used for early cancer screening, prognostic monitoring, and other potential point-of-care applications in the future.

## 4. Breast Cancer Dormancy

Quiescence and senescence are both dormant cellular states and, although distinct, they are frequently used as interchangeable terms in literature unwittingly. Senescence is the gradual deterioration of functional characteristics in living organisms, a phenomenon characterized by the cessation of cell division, whereas quiescence is reversible to proliferation upon growth signals [193]. Cell quiescence, viewed as dormancy with minimal metabolic activity, occurs due to a lack of nutrition and growth factors, whereas senescence takes place due to aging and serious DNA damages. Hence, in contrast to quiescence, senescence is a degenerative process ensuing certain cell death. The reactivation of quiescent cells upon physiological growth signals and the reversal of quiescent cells to proliferation are fundamental to many physiological phenomena, such as tissue homeostasis and repair [194,195,196]. More recent studies have indicated that quiescence is not a passive existence, but rather an actively maintained and highly heterogeneous cell state [197,198]. It has been widely accepted that cell quiescence heterogeneity can be described as graded depth [199,200]. The dysregulation and loss of quiescence often results in an imbalance in progenitor cell population, ultimately leading to stem cell depletion, and the in vivo heterogeneous re-entry to cell cycle from quiescence is beneficial by avoiding complete deprivation of the quiescence pool [201].

### 4.1. Quiescence in Breast Cancer

Quiescence in cancer cells is considered a major therapeutic challenge because it confers dormancy in tumor, hence circumventing inherent anti-neoplastic surveillance system and standard-of-care cancer therapeutics including chemotherapy and radiotherapy. In the clinic, tumors are considered dormant if they recur after at least 5 years from surgical treatment and, in patients with breast cancer, recurrence can take place even after decades of apparent disease-free survival [202]. Whether tumor dormancy reflects the presence of quiescent cancer stem cells (CSCs) among the cells disseminated from the primary cancer is still not clear. However, because the quiescence phenomenon is actively regulated by various intrinsic mechanisms rather than a passive state; hence, it has been inferred that the quiescent or dormant CSCs are also able to perceive signal changes from its microenvironment and, thus, responding by activating its machineries to re-enter the cell cycle. In the cancer context, mTOR activation is a critical regulators to enhance the proliferation and colony formation capability of several CSC types, including breast. Hence, inhibition of the mTOR pathway could possibly attenuate the proliferative capacity of these CSCs, leading them to remain in their quiescent state [203]. MDA-MB-231 and T47D breast cancer cells have been shown to enter a quiescent state with reduced proliferative activity upon receiving exosomes released by bone marrow stromal cells that contain CXCL12-specific miRNAs [204]. Breast cancer cells were found to intercalate into the hepatic niche without interfering with hepatocyte function in a human ex vivo hepatic microphysiologic system. The microfluidic system was established with fresh human hepatocytes and non-parenchymal cells (NPCs), creating a microenvironment into which breast cancer cells (MCF7 and MDA-MB-231) were added. Examination of the cancer cells demonstrated that a significant subset enters a quiescent state of dormancy, shown by a lack of cell cycling. The presence of NPCs altered the cancer cell fraction entering quiescence, and led to differential cytokine profiles in the microenvironment effluent [112]. An in vitro model of tumor dormancy and recurrence has been established using the drug resistance mode, in which breast tumor cell lines were exposed to short-term chemotherapy drugs at clinically relevant doses. As a result, the dormant, chemoresistant, and slow cycling tumor cells—or presumably CSCs—were enriched [205]. It has also been revealed that dormancy characteristics, including reduced proliferation rates and stem-cell-like surface markers, can be induced in metastatic breast cancer cells by co-culturing them with exosome-containing miR-23b isolated from bone-marrow-derived mesenchymal stem cells (MSCs) [206]. Additionally, breast cancer cells were observed to activate MSCs for the release of exosomes containing miRNAs, which stimulate quiescence in a subpopulation of cancer cells that are heightened with drug resistance nature [207]. These studies, therefore, indicate an importance of extracellular stimuli for the cancer stem cells to achieve a quiescent state, and hence represent a potential cancer therapy approach to block this mode of communication. Most therapeutics target actively proliferating cancer cells; thus, these cells eventually develop a quiescent nature as a mechanism of survival and progression under both niche and therapeutic pressures. The quiescence state in cancer cells confers resistance to conventional cancer therapies, leading to disease progression and relapse. Therefore, targeting quiescent cancer cells or CSCs is a promising therapeutic approach; however, extensive research is still needed to devise an effective therapy [208].

### 4.2. Microfluidics for Quiescence Research

Understanding the mechanisms governing the chemoresistance of individual stem cells may require analysis at the single cell level. This task is not trivial using current technologies, given that isolating stem cells is difficult, expensive, and inefficient, due to low cell yields from patients. Particularly, hematopoietic cells are largely non-adherent and thus difficult to study over time using conventional cell culture techniques. Hence, there is a need for new platforms that allow the functional interrogation of hundreds of single cells in parallel. Although there has been a growing interest in the research community, the full potential of microfluidics in studying cell dormancy and quiescence has yet to be realized. The ability to perform assays within a microfluidic platform has been demonstrated using minimal reagents and low numbers of primary cells. Investigating normal and chronic myeloid leukemia (CML) stem cell responses to the tyrosine kinase inhibitor, it was hypothesized that this disease persistence resulted from a population of quiescent CML stem cells which are resistant to therapy [209]. Heterogeneity in cell populations poses a major obstacle to understanding complex biological processes, and there is a pressing need for the scalable analysis of single cells. Analyses of clonal cultures established from single cells have previously provided many insights, including the observation that quiescence and delayed cell-cycle entry correlate with higher self-renewal potency. Thus, exploiting the high-throughput potential of microfluidic systems to investigate proliferation control at the single-cell level, a platform containing thousands of nanoliter-scale chambers has been proposed for the live-cell imaging interrogation of clonal cultures of nonadherent cells, with precise control of the conditions for the in situ immunostaining and recovery of viable cells [210]. A microfluidics method has been developed to enrich physically deformable cells by mechanical manipulation through artificial micro-barriers. Using breast cancer cell lines under mammosphere culture conditions, rare quiescent and slowly dividing cells were reported to retain PKH26 fluorescence, although rapidly growing cells lose fluorescence by dilution with each proliferative cycle [211]. The characterization of single-cell metabolism has been recognized as imperative for understanding subcellular functional and biochemical changes associated with healthy tissue development and the progression of numerous diseases, including cancer. A robust assay using droplet microfluidic technology, developed to study cell heterogeneity within and among cell lines, has been extended to characterize metabolic differences between proliferating and quiescent cells—a critical step toward label-free single cancer cell dormancy research. This noninvasive droplet-based and label-free method, using an expansion flow-focusing geometry to distinguish individual cells based on their metabolic states, could therefore be used as an upstream phenotypic platform to correlate with genomic statistics [212]. The non-proliferative cellular quiescence state is required for cell survival under stress and during development. In most quiescent cells, proliferation is stopped in a reversible state of low Cdk1 kinase activity; in many organisms, however, quiescent states with high Cdk1 activity can also be established through still uncharacterized stress or developmental mechanisms. A microfluidics approach has been utilized to identify stress pathways associated with starvation-triggered high-Cdk1 quiescent states in cells. The choice between low- or high-Cdk1 quiescence was found to be controlled by the cell-cycle-independent accumulation of Xbp1, which acted as a time-delayed integrator of the duration of stress stimuli. The results indicate how cell-cycle-independent stress-activated factors promote cellular quiescence outside G1/G0 [213]. Stem cell behavior is extremely sensitive to environmental stimuli, which are difficult to manipulate, demonstrate, and quantify with traditional methods. The stem cell niche is a reservoir of multipotent stem cells that can maintain normal, injured, or aged organs and tissues, in response to signals that regulate whether they should remain quiescent, undergo self-renewal, or differentiate. Researchers have recently created microfluidic microenvironments that can emulate several key properties of the stem cell niche in vitro to enable reductionist studies of their influences on stem cell behavior, including both biochemical and biophysical regulation [214]. The balance between cell quiescence and proliferation is fundamental to tissue physiology, and the activation of quiescent cells to proliferate is critical for tissue homeostasis and repair. Recognizing the role of interstitial fluid flow in the cellular microenvironment, a microfluidic platform was used to elucidate the effect of fluid flow on cellular quiescence depth. Indeed, the results indicate that extracellular fluid flow alters cellular quiescence depth through flow-induced physical and biochemical cues, which could help better understand the heterogeneous response of quiescent cells for tissue repair and regeneration in the physiological context of living tissues [215].

## 5. Breast Cancer Therapeutic Development

### 5.1. Drug Development and Delivery

**Figure 7:**
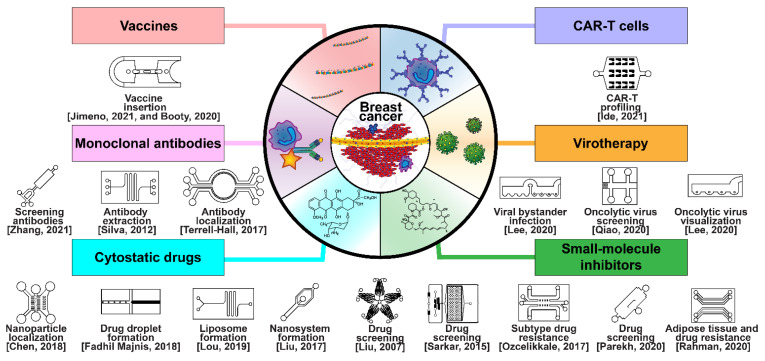
Selected microfluidic platforms constructed to aid the development of modern drug and biological anticancer treatments of breast cancer, which are classified in six modalities: cytostatic drugs, small-molecule inhibitors, monoclonal antibodies (MAbs), chimeric antigen-specific receptor transfected T cells (CAR-Ts), virotherapy, and vaccine therapy.

Breast cancer has been treated with pharmacologic therapy for nearly a century, with biological therapies becoming more popular in recent decades [17]. Modern drug and biological anticancer falls into six categories: cytostatic drugs, small-molecule inhibitors, monoclonal antibodies (MAbs), chimeric antigen-specific receptor-transfected T cells (CAR-Ts), virotherapy, and vaccine therapy (Figure 7) [17]. Cytostatic drugs, such as cyclophosphamide, doxorubicin, and paclitaxel, are the most common chemotherapy drugs in breast cancer, and are often used in combinations [216,217]. A focal theme in cytostatic drugs is the CDK4/6 cell-cycle checkpoint proteins, which are inhibited by ribociclib, palbociclib, and abemaciclib [218,219,220]. The three compounds have already been approved by the U.S. FDA for use together with endocrine therapy. Small-molecule inhibitors are used in targeted therapy to interfere with specific mechanisms in cancer metastasis, such as sirolimus, which inhibits mTOR expression, and copanlisib, which inhibits PI3K [221]. Monoclonal antibody chemotherapy involves the injection of antibodies that locate and disrupt receptors associated with cancer, such as ErbB-2-targeting trastuzumab and EpCAM/CD326-targeting adecatumumab [222,223]. Some MAbs are conjugated with toxins to localize them in tumors, whereas others conjugate tumor cells to nearby immune cells [224]. CAR-T cells are personalized, genetically modified, anti-tumor T cells that have engineered pathways such as internalized T cell activating signaling proteins such as 4-1BB, OX40, and ICOS, and which may only activate through external anti-tumor antibody antigen attachment [225,226,227]. Viral therapy is classified into viroimmunotherapy or oncolytic virotherapy; viroimmunotherapy uses reverse transcription through lentivirus or adenovirus to transfect T neutrophils with anti-cancer antigens, whereas oncolytic virotherapy directly upregulates apoptotic pathways within tumors [228,229]. Finally, anti-cancer vaccines prime T neutrophils to attack breast cancers by antagonizing particular receptors, such as the HER2/ERbB [230].

Microfluidic technology is very useful for repeatably handling complex fluids, and as such, has been used to investigate and manufacture treatments for breast cancer. Microfluidic platforms for drug screening provide rapid and repeatable results, and have been applied to investigate treatment impacts at every stage of the breast cancer metastatic cascade [231,232]. The most significant advantage of microfluidic over traditional approaches is the ability to recapitulate the tumor microenvironment through the precise control of physiological cues such as hydrostatic pressure, shear stress, oxygen, and nutrient gradients. An HMEpiC/MDA-MB–231 co-culture system was established to model mild, moderate, and severe cancer for investigating cancer cell migration and anti-cancer drug screening [74]. The application of a microfluidic platform for drug discovery and personalized medicine has been demonstrated by analyzing the response to chemo- and anti-angiogenic therapy [77]. A 3D microfluidic breast cancer tissue model has been utilized for evaluating the photodynamic therapy of breast cancer, which produces reactive oxygen species near specific molecules when exposed to a particular wavelength of light [233]. Breast cancer spheroids formed in a microfluidic device were exposed to pH-sensitive nanoparticles, which encapsulated anti-cancer drugs with surface-bound folic acid, to induce TNBC-specific phagocytosis [234]. To investigate the EPR effect, a secluded breast cancer tumor tissue in a microfluidic device was exposed to dual antibody-treated liposomes as a drug delivery mechanism [23].

Several reviews have described different microfluidic diagnostic and drug delivery systems (DDSs) discussing tumor models proposed for the characterization of delivery, efficacy, and toxicity of cancer nanomedicine, as well as the impact of the microfluidic device design and parameters on the type and properties of DDS formulations [235,236,237]. In general, whereas some systems focus on the investigation of how drugs behave when introduced into microfluidic tumor models, other systems concentrate on the creation of encapsulated or drugs prepared otherwise using microfluidics [238]. Drug preparation techniques using microfluidic systems include vesicle, liposome, or micelle encapsulation, polymeric nanocrystaline or lipid nanoparticle dispersion, and multilayered microspherical encapsulation [239,240,241,242,243].

Biological therapeutics such as MAbs, CAR-T, vaccines, and virotherapy have also been investigated in microfluidic systems, where some devices were utilized to manufacture, harvest, and sequence MAbs [244,245,246,247]. The anti-ErbB-2 MAb trastuzumab has been prepared with radioactive ^89^Zr for PET scanning in a microfluidic device, and the localization of trastuzumab to distal tumors was modeled in a similar device [248,249]. Rapidly screening for MAbs of surface receptors on breast cancer has been carried out in a microfluidic platform for use in clinical analysis [250]. The microfluidic purification of T lymphocytes separated from blood for chimeric antigen receptor T cell manufacturing has been demonstrated for CAR-T cell therapy reinjection [251], and the gene editing required to manufacture CAR-T cells has also been mechanized by microfluidic devices incorporating electroporation for CRISPR/CAS-9 modification [252]. Profiling T cell interaction and activation through serial encounters with antigen-presenting cells (APCs) has been accomplished in a microfluidic device mimicking the microenvironment of a lymph node [253]. Anti-cancer vaccines have also been under investigation using microfluidic platforms, such as the CellSqueeze commercial system, incorporating microchannels to squeeze and stretch cells for permitting rapid vaccine delivery [254,255]. Finally, anti-cancer virotherapy investigations have utilized microfluidic devices to monitor oncolytic viruses in real time, study bystander infection, and localize viruses according to the pH level [256,257,258,259,260].

### 5.2. Cancer Resistance to Treatment

Resistance to targeted therapies is a major clinical challenge in cancer treatment. Despite technological advances, robust biomarkers or platforms predictive of treatment responses are lacking due to the inherent nature of complex genomic landscape of cancer. Exploring breast cancer chemotherapy resistance is very complicated due to the disparate mechanisms by which tumors engage with external signaling. For instance, breast cancer develops resistance to cytostatic doxorubicin through the modulation of matrix proteins, adaptive apoptosis suppression through p53, and EMT to increase motility and escape the environment [261,262,263,264]. Some of these adaptive resistances to doxorubicin can be tied to a small amount of shared signaling proteins such as the MAPK signaling pathway but, in general, resistance can only be investigated using next-generation sequencing techniques to identify transcriptomic signatures associated with chemotherapy-resistant breast cancer [265,266,267]. Biological chemotherapies are less susceptible to resistance, but CAR-T targets may downregulate antigens such as CD19/20, vaccines can lose ecto-CRT/CALR surface receptors, and viruses may lead to bottlenecks that enable non-vaccinated to replace the vaccinated tumors [268,269,270].

Recent efforts are centered on performing direct drug screening on patient-derived cells through their ex vivo expansion and maintenance to facilitate the personalized stratification of treatment modalities. Microfluidics is a technology well suited for chemotherapy resistance screening due to its high degree of control, enabling high-throughput drug screening through parallelization and automation using small-volume samples. Parallel microfluidic networks have been developed for studying the cellular response to chemical modulation by exposing cells to gradients of up to five separate therapies and capturing them for imaging to analyze their adaptive response [271]. A droplet microfluidics-based approach was presented to assess the dynamics of drug uptake, efflux and cytotoxicity in drug-sensitive and drug-resistant breast cancer cells [272]. Differential responses to doxorubicin in breast cancer subtypes were simulated by a microfluidic tumor model, and a method of overcoming doxorubicin resistance using a hyaluronic nanoparticle encapsulation was developed [273]. Anti-cancer drug efficacy was assessed in a tumor-mimetic platform designed to recreate the microvasculature of high- and low-perfusion breast cancer tumors, which were treated with small-molecule drugs, and low-perfusion tumors were found to be sheltered from anti-cancer drugs despite the EPR effect [20]. Starvation-induced spatial–temporal metabolic adaptations were explored in an organotypic microfluidic breast cancer model, revealing that heterogeneous adaptations to metabolic stress in nascent tumors make anti-cancer drugs targeting cancer metabolism less effective [21]. A microfluidic device, developed for the rapid isolation of exosomes produced by multiple drug-resistant cancer cells in response to various therapies, was used to identify the mechanisms of adaptive resistance [274]. Measurements of multidrug resistance in single breast cancer cells, captured in a microfluidic chip, allowed the automated isolation and purification of chemotherapy-resistant drugs [275,276], and crosstalk pathways between breast cancer cells and adipose-derived stem cells that lead to drug resistance were identified via passive diffusion in a two-layer microfluidic device [277]. Thus far, several microfluidic platforms have been successfully applied for the maintenance and expansion of patient-derived tumor cells, spanning diverse cancer types and sources, solid tumors or liquid biopsies (CTCs), for personalized drug screening applications [232].

## 6. Summary

Microfluidic technology has enabled the reconstitution of functional units of organs on chips, far superior to traditional cell culture or animal models, to investigate human diseases such as cancer, with breast cancer being the second leading cause of cancer death among women. Table 1 summarizes the key features of selected microfluidic platforms specifically developed for breast cancer research. Metastasis is a key event of cancer progression and the primary cause of mortality in breast cancer patients. Breast cancer cells may metastasize through axillary lymph nodes or systemic circulation, with the latter being the dominant route. Despite the clinical importance of breast cancer metastases, early research has largely focused on the oncogenic transformations leading to the development of primary tumors, and much remains to be learned about the metastatic process. Consequently, breast cancer metastasis is now a major field of research in oncology, and microfluidics has been making steady contributions to this field. Microfluidic-based models have shown great potential in investigations of the breast cancer metastasis cascade by providing 3D constructs mimicking the in vivo cellular microenvironment coupled with the ability to monitor cellular interactions in real time. In order to enhance the physiological and clinical relevance of microfluidic models, human primary cells are gradually replacing the immortalized cancer cell lines, incorporating different cell types to more faithfully recapitulate the complex in vivo paracrine signaling that regulates breast cancer metastasis and organotropism. Moreover, new projects are under way, integrating on-chip advanced proteomics and genomics analysis, which have the potential to accelerate research into other critical domains of breast cancer such as early detection, therapeutic development, treatment efficacy, disease monitoring, and recurrence. Microfluidics may prove to be a key technology not only for basic research, deciphering fundamental biochemical mechanisms involved in breast cancer, but also for clinical applications towards personalized medicine.

## Figures and Tables

**Table 1:** Summary of selected microfluidic systems used in breast cancer research.

Reference	Cell Used	Culture Type	Field of Investigation	Device Properties	Findings
Cheng [19]	Acellular	2D	Cancer formation	Extract cell-free DNA from plasma to detect BRCA1 and BRCA2 mutations.	Successful detection of BRCA1/2 mutations with a minimum detectable number of copies of 20,000.
				Uses four distinct primers in parallel to provide point of care risk assessment.	
				Dimensions not described, no ECM used, utilizes pressure differential pumping.	
Pradhan [20]	MCF7 and MDA-MB-231 cancer, hBTEC endothelial, BJ5ta fibroblast	3D	Cancer formation	Two distinct chips with normo- and pathophysiologic vascular layout, respectively, used to assess anti-cancer drug delivery and tumor reaction to treatment.	Cells found to elongate and align along flow, dependent on the cell line.
				Model of cancer–stromal–endothelial interactions within a pillar-filled tumor region adjacent to vessels.	MCF7 found to have significant resistance to anti-cancer drug paclitaxel in low-perfusion chip design.
				100 μm channel width, PEG–fibrinogen hydrogel matrix, high perfusion layout experiences 40–50 s^−1^ shear rate, low perfusion chip experiences 10–20 s^−1^ shear rate.	Both MCF7 and MDA-MB-231 found to have significant resistance to anti-cancer drug doxorubicin in low-perfusion chip design.
Ayuso [21]	MCF10A cancer, HMF fibroblasts	3D	Cancer formation	Ductal carcinoma in situ in a central vessel surrounded by stroma-filled matrix between two empty channels for perfusion, metabolism, mobility, and gene expression investigation.	Hypoxia-activated Tirapazamine selectively destroys DCIS cells.
				Breast cancer cells, fibroblasts, and hydrogel model of luminal mammary duct.	Hypoxia-activated glycolysis transcriptome upregulation.
				Dimensions not described, collagen hydrogel, static conditions with daily media change.	
Funamoto [22]	MDA-MB-231 cancer	3D	Cancer formation	Five-channel chip with central tumor model, surrounded by two media perfusion channels, which are, in turn, surrounded by two gas perfusion channels.	Hypoxia found to significantly increase cancer mobility.
				Breast cancer cells in 3D culture with controlled oxygen perfusion to investigate hypoxia effects.	
				1.3 mm tumor channel width, 0.5 mm media and gas channel widths, collagen I hydrogel, 30 μL/h flow.	
Tang [23]	MDA-MB-231 and MCF-7 cancer, primary human-breast-tumor-associated endothelial cells	3D	Cancer formation	Microfluidic chip modeled on 2D projection of tumor vasculature to investigate enhanced permeability and retention (EPR) found in tumors.	TNF-α found to significantly increase the permeability of endothelial cells.
				Breast cancer and endothelial cell biomimetic tumor microenvironment.	Tumor cell co-culture significantly increases the permeability of endothelial cells.
				100 μm channel width, fibronectin ECM, 60 uL/h or 0–90 s^−1^ shear rate.	Liposome extravasation through endothelial cells found to significantly increase during tumor co-culture.
Nashimoto [24]	MCF-7 and GFP MDA-MB-231 cancer, RFP HUVECs, normal human lung fibroblasts, SW620 with luciferase, Hepg2	Spheroids	Cancer formation	2 or 3 cell type co-culture spheroids of various cancers in 96-well plate transferred into microfluidic chip for angiogenesis.	Fibroblast co-culture induced angiogenic sprouts.
				1 mm width culture channel, fibrin-collagen matrix, 30 μL/h.	Flow reduces anti-cancer drug paclitaxel efficacy and leads to less necrotic tumors.
Uliana [32]	Acellular	2D	Cancer formation	Disposable microfluidic electrochemical array device to detect estrogen receptor alpha (ERα).	USD 0.20 cost of manufacture.
				Highly decorated magnetic particles and protein–DNA interaction detected using electrodes to quantify cancer signals.	Ultralow detection limit of 10.0 fg mL^−1^ for the determination of ERα in calf serum.
				3 mm wide and 200 mm long, no ECM, 6000 μL/h.	Good recoveries for detection of the biomarker in MCF-7 cell lysate.
Moon [44]	MCF-7, MDA-MB-231, and SUM-159PT cancer	3D	Cancer formation	Hydrogel tube seeded with breast cancers to quantify breast cancer subtype motility.	SUM-159PT found to be most invasive cell line.
				500 μm diameter, collagen I hydrogel, static conditions.	CD24 expression was elevated in 3D compared with 2D cultures.
Yong [45]	SUM149, HCC1937, MDA-MB-231, and BT549 cancer	3D	Cancer formation	A hydrogel channel with two perfused vessels, one seeded with breast cancer cells to quantify the directional migration of cancer cell lines.	MDA-MB-231 found to be most invasive.
				200 μm diameter, collagen hydrogel, static conditions.	305 genes identified as altered during invasion.
Wang [251]	SK-BR-3 cancer, HEK293FT cells, human T cells, Jurkat cells	Suspended cells	Cancer formation	A pair of chips, one that generates droplets containing cells and antibodies or lentivirus, another that electrically sorts droplets based on fluorescence.	CD40 antibody developed.
				Continuous flow droplet-based lentivirus transduction and antibody screening.	Active anti-Her2 × anti-CD3 BiTE antibodies developed using antibody library.
				40 μm deep channels, no ECM used, 600–1800 μL/h.	
Sung [46]	MCF-DCIS.com cancer cells, GFP Human mammary fibroblasts	3D	Invasion	Mammary endothelial and fibroblast cells co-cultured in Y-shaped compartmentalized model.	Co-culture promotes invasion, diminishing as distance between cell types increases
				Second harmonic collagen imaging provides an index of transition to invasion.	Soluble factors shown to begin migration, while cell–cell contact between cancer and stromal cells completed the transition to invasion.
				1.5 mm width, Matrigel and collagen I matrix, static culture.	
Lam [47]	RFP MDA-MB-231, skin fibroblasts	3D	Invasion	Two parallel 3D models with breast cancer and fibroblast co-cultures to study the role of acidity in the transition to invasion.	Calcium bicarbonate buffer nanoparticles inhibit acid-induced invasion.
				Channels between 200–1000 μm wide, fibrin hydrogel, 36 μL/h	Buffering selectively inhibited the growth of the MDA-MB-231.
Toh [48]	MX-1, MCF7 cancer	3D	Invasion	Tumor cells encased in collagen exposed to flowing chemoattractant signals on either side.	Cells found to exhibit collective, amoeboid, and mesenchymal-like motility.
				Real-time monitoring of cell extravasation	Distinct populations of collagen-penetrating invasive cancer cells and collagen-avoidant migratory cancer cells identified.
				1 cm long, 600 μm wide, and 100 μm tall, positively charged collagen and a negatively charged HEMA-MMA-MAA terpolymer matrix, 30 μL/h.	
Yankaskas [49]	ZR75-1, MDA-MB-468, MDA-MB-436, Hs578t, BT-549, and MDA-MB-231 cancer cells, MCF-10A, T47, and human mammary epithelial cells, and HCC1428	3D	Invasion	Continuous flow of breast cancer cells near parallel apertures through which chemoattractants diffuse to induce migration.	Identified motility- and survival-related genes.
				Quantifies extravasation potential, abundance, and proliferative index of breast cancer cell subtypes.	MDA-MB-231 found to be most migratory.
				Width of 20 µm and a height of 10 µm, collagen matrix, flow not specified.	Self-sorted migratory cells found to be significantly more proliferative and preferentially localize to bone in comparison with non-sorted cells.
Gioiella [50]	MCF7 cancer cells, normal and cancer activated fibroblasts	3D	Invasion	Breast cancer tumor model using epithelial and stromal cell co-culture.	Hyaluronic acid and fibronectin overexpression are associated with invasion
				Macromolecule and collagen production quantified.	Fibroblasts significantly lose diffusivity when activated by nearby tumor cells.
				370 μm long, 780 μm wide, 300 μm deep, other channel is 1200 μm long, 1370 μm wide, 300 μm deep, crosslinked type A porcine-derived gelatin matrix, 180 µL/h.	
Mosadegh [51]	A549	3D	Invasion	Stacked paper seeded with hydrogel and cancer cells, used to examine hypoxia-induced migration.	Oxygen acts as a chemoattractant for cancer cells.
				No channels, Matrigel matrix, no perfusion.	Distinct rates of oxygen chemotaxis between cell lines.
Lugo-Cintron [53]	MDA-MB-231 cancer cells, normal and cancer activated fibroblasts	3D	Invasion	Large channel containing fibroblasts in microenvironment with a central channel seeded with breast cancer cells.	Fibronectin-rich matrix associated with increased migration.
				3D breast cancer and fibroblast tumor model to visualize invasion.	MMP secretion associated with increased migration.
				400 μm wide central channel, rat-tail collagen type I, fibronectin, and fibrin matrix, static culture.	
Buchanan [72]	MDA-MB-231 cancer cells, telomerase immortalized microvascular endothelial (TIME) cells	3D	Intravasation	Microchannel embedded within a collagen hydrogel as a vessel model with shear quantified using microparticle image velocimetry.	Tumor cells significantly increase the expression of proangiogenic genes in response to co-culture with endothelial cells under low flow conditions.
				Microfluidic tumor vascular model for co-culture of tumor and endothelial cells under varying flow shear stress conditions	Endothelial cells develop a confluent endothelium on the microchannel lumen that maintains integrity under physiological flow shear stresses.
				850 μm diameter, Collagen 1 matrix, 180 μL/h.	
Choi [73]	Human primary mammary fibroblasts, HMT-3522 cells	Spheroids, 3D	Intravasation	Intravasation model using a two-channel stroma and endothelial co-culture device later seeded with human mammary ductal epithelial cells and mammary fibroblasts co-culture breast tumor spheroids.	Paclitaxel anti-cancer drug shown to inhibit progression through cytotoxicity.
				Visualization of attachment and intravasation.	
				1 mm wide, 3 mm long, and 200 μm tall, collagen hydrogel, 60 μL/h.	
Nagaraju [76]	MDA-MB-231 and MCF7 cancer cells, HUVECs	3D	Intravasation	Chip with distinct tumor, stroma, and vascular channels to model invasion and intravasation into media-filled vascular channel.	Endothelial cells significantly increase the migration of tumor cells.
				200 μm wide channel, collagen matrix, flow rate not specified.	Tumor signaling significantly reduces vessel diameter and increases endothelial permeability.
					Absence of endothelial cells significantly alters the secretion of ANG-2 and angiogenin.
Zervantonakis [71]	MDA231 cancer cells, HT1080 fibroblasts	3D	Intravasation	Three-channel tumor model with an endothelial channel and a tumor channel separated by a cell-free 3D ECM channel to model intravasation towards endothelial cells.	Macrophage-secreted tumor necrosis factor alpha significantly increases intravasation rate.
				500 μm wide, 20 mm in length, and 120 μm in height, collagen matrix, flow not specified.	Endothelium provides a barrier to intravasation, regulated by tumor microenvironment factors.
Cui [75]	MDA-MB-231 cancer cells, primary human vascular endothelial cells	3D	Intravasation	32 independent cell collection microchambers with endothelial layer for characterization of trans-endothelial migration.	Migratory cancer cells significantly alter Palladin expression, F-actin orientation, and cell aspect ratio.
				4 mm by 4 mm chip, poly-D-lysine and fibronectin matrix, 1200 µL/h.	Different cancer lines show significantly distinct sensitivity to shear stress impact on trans-endothelial migration
Shirure [77]	MDA-MB-231 and MCF-7 cancer cells, Endothelial colony forming cell-derived endothelial cells, normal human lung fibroblasts, colorectal cancer cell line Caco-2	3D and Spheroid	Intravasation	Two tumor model chambers separated by one fibroblast and endothelial cell vascular model chamber to model arterial capillary intravasation.	VEGF and TGFβ significantly elevated during intravasation and migration.
				355 μm wide, fibrin matrix, 10 mm H_2_O pressure head.	Tumor cells expressing mesenchymal-like transcriptome invade into vascular chamber significantly more efficiently.
Jiang [146]	Lung, breast, and melanoma cancer blood samples	Suspended cells	Circulating signals and cells	Chip using deterministic lateral displacement to enrich platelet-CTC aggregates in conjugation with platelet antibodies for isolation.	60% reliable isolation of breast cancer CTC clusters.
				24 parallel channels that are 150 μm in depth, no ECM, 1000 μL/h.	
Au [147]	Primary breast cancer CTCs and CTC clusters	Suspended cells	Circulating signals and cells	Two-stage continuous isolation of CTC clusters using asymmetry induced rotation.	2–100+ cells recovered from whole blood.
				99% recovery of large clusters, cell viability over 87%.	
Deliorman [149]	PC3 human prostate cancer cell line	Suspended cells	Circulating signals and cells	Surface-bound antibodies for EpCAM, PSA, and PSMA to isolate CTCs for AFM measurements.	CTCs from metastatic cancer have decreased elasticity and increased deformability compared with tumor cells.
				900 μm wide, 85 μm deep, and 48 mm long, no ECM, 1200 μL/h.	Fewer multiple adhesion events in CTCs compared with tumor cells.
Sarioglu [158]	MDA-MB-231	Suspended cells	Circulating signals and cells	Detection of CTCs independent of tumor-specific markers using bifurcating traps under low-shear conditions.	30–40% successful detection of CTC clusters.
				4096 parallel 60 μm wide traps, no ECM, 2.5 mL/h.	RNA sequencing identifies macrophages within CTC clusters are tissue-derived from the primary site.
de Oliveira [163]	Acellular	Suspended cells	Circulating signals and cells	CTC detection using double-layer capillary capacitors to quantify CA 15-3.	5µL sample volume and 92.0 μU mL^−1^ detection limit.
				Antibody-anchored magnetic bead capture of CTCs for analysis.	USD 0.97 cost of manufacture.
				800 μm outer diameter, 545 μm inner diameter, 200 μm electrode gap, no ECM, no flow.	
Marrella [159]	MDA-MB-231	Suspended cells	Circulating signals and cells	Multi-channel device simultaneously analyze correlation between shear stress and CTC cluster behavior.	Higher values of wall shear stress significantly correlated with decreased CTC viability to metastasize.
				1 mm wide and 120 mm long, no ECM, 0–20 dyne/cm^2^.	High shear stress significantly disaggregates CTC clusters within 6 h.
Regmi [160]	MDA-MB-231 and UACC-893 cancer cells, lung cancer A549, ovarian cancer 2008, leukemia K562 cells	Suspended cells	Circulating signals and cells	Microfluidic circulatory system to produce relevant shear stress levels on CTCs and CTC clusters to investigate disaggregation under shear.	60 dynes/cm^2^ during exercise leads to necrosis or apoptosis in 90% of CTCs after 4 h of circulation, significantly more than 15 dynes/cm^2^.
				1mm diameter tube, no ECM, 15–60 dyne/cm^2^.	High shear significantly reduces metastatic potential and drug resistance of breast cancer cells.
Uliana [32]	MCF-7 cells, a human breast cancer cell line	Suspended cells	Circulating signals and cells	Disposable microfluidic electrochemical arrays using electrode-bound DNA and antibody-conjugated magnetic beads to detect estrogen receptor alpha in plasma.	10.0 fg mL^−1^ detection limit.
				1.5 mm diameter electrode, no ECM, 6000 μL/h.	94.7–108% recovery of estrogen receptor alpha.
					USD 0.20 cost of manufacture.
Vaidyanathan [187]	BT-474 and MDA-MB-231 cancer cells, PC3 prostate cancer cells	Suspended cells	Circulating signals and cells	Multiplexed electrohydrodynamic detection of exosome targets in a chip.	Isolation of exosomal samples from HER2 and prostate-specific antigen.
				400 μm wide, 300 μm tall, 25 mm length, no ECM, 420 μL/h.	8300 exosomes/µL detection sensitivity.
Gao [191]	Acellular	Suspended cells	Circulating signals and cells	Multiple detections of miRNAs from multiple samples using three-segment hybridization detection in a microfluidic chip.	Successful detection of four breast cancer biomarker miRNAs.
				50 independent channels on a 25 by 75 mm^2^ chip, no ECM, flow not described.	Detection limit of 1 pM in 30 min.
Armbrecht [110]	MCF-7, SK-BR-3, and BR16 cancer cells, LM2 variant cell line	Suspended cells	Organotropism and extravasation	Integrated capture, isolation, and membrane analysis of CTCs in a chip.	95% isolation efficiency, granulocyte growth-stimulating factor detection efficiency at 1.5 ng/mL^−1^.
				30 independent 75 μm width chambers, no ECM, 12 μL/h.	
Park [111]	MCF-7 and MDA-MB-231 cancer cells, HL-60 promyelocytic leukemia cell line	Suspended cells	Organotropism and extravasation	Inertial-force-assisted droplet generation using spirals to generate CTC clusters with known cell type ratios.	E-cadherin, VCAM-1, and mRNA expression characterized between cluster compositions.
				140 μm width and five-loop structure, no ECM, 1200 μL/h.	
Riahi [113]	MDA-MB-231 cancer cells, HUVECs, human mammary epithelial cells, MCF7	Suspended cells, 3D	Organotropism and extravasation	Organ-specific extravasation of CTCs through an endothelial layer using localized chemokine gradients in a chip.	CXCL12 found to significantly increase extravasation.
				150 μm by 500 μm by 3 cm, Matrigel matrix, 50 μL/h.	
Aleman Sarkal [114]	HUVECs, HCT-116 colorectal cancer, HEPG2, A549	Suspended cells, 3D	Organotropism and extravasation	Organ-specific extravasation of CTCs modeled using bioengineered 3D organoids of five different tissues.	HCT115 CRC cells preferentially extravasate into liver and lung cells, as seen in vivo.
				200 μm width, HA/gelatin matrix, 600 μL/h.	
Song [115]	MDA-MB-231, MCF-10A, and MCF-7 cancer cells, HUVECs, normal human lung fibroblasts	Suspended cells, 3D	Organotropism and extravasation	Microvascular network model to quantify the extravasation of breast cell lines in distinct oxygen conditions.	HIF-1a confirmed through knockout and siRNA to significantly increase the transmigration capacity in breast cell lines and regulate apoptotic-related cellular processes.
				1 mm wide and 150 µm deep, fibrin matrix, static conditions.	In hypoxia, HIF-1a levels increased alongside changes in morphology and an increase in cancer viability and metastatic potential.
Marturano-Kruik [116]	GFP MDA-MB-231, Firefly MDA-MB-231, HUVECs, human bone marrow mesenchymal stem cells	3D	Organotropism and extravasation	Perfused bone perivascular niche on a chip to measure progression and drug resistance during metastasis.	Bone-marrow-derived mesenchymal stem cells shown to transition towards perivascular cell lineages and support capillary formation.
				Dimensions not shown, decellularized bone ECM matrix, 15 μL/h.	Interstitial flow within bone perivascular niche persists with low proliferation and high drug resistance.
Jeon [117]	MDA-MB-231 cancer, GFP HUVECs, human bone marrow mesenchymal stem cells	3D	Organotropism and extravasation.	Microfluidic bone, vascular, and myoblast model to analyze breast cancer organotropism.	Extravasation rate and permeability found to be significantly distinct between bone, myoblast, and unconditioned matrix models.
				1.3 mm wide and 200 μm deep, fibrin ECM, 120 μm/h.	A3 adenosine receptor disruption resulted in significantly higher extravasation rates to myoblast-containing matrix
Bersini [118]	GFP MDA-MB-231 cancer, RFP HUVECs, human bone marrow mesenchymal stem cells	3D	Organotropism and extravasation	Osteo-differentiated bone marrow-derived mesenchymal stem cells and endothelial cell bone microenvironment model of organotropism of breast cancer CTCs in a chip.	CTCs extravasated into the bone model 77.5% of the time at a distance of 50.8 µm, compared with 37.6% of the time at a distance of 31.8 µm into collagen control.
				Eight parallel 225 µm by 150 µm gel regions, collagen hydrogel ECM, static conditions.	Bone secreted signals CXCR2 and CXCL5 found to influence extravasation rate and travel distance.
Mei [119]	MDA-MB-231 cancer cells, HUVECs, MLO-Y4 osteocytes, RAW264.7 osteoclasts	3D	Organotropism and extravasation	Breast cancer, endothelial cell, and osteocyte-like cell model of bone extravasation using oscillatory shear in a chip.	3.71-fold increase in calcium response in 82.3% of osteocytes compared to continuous flow.
				1 mm by 200 μm static channel in addition to a 500 μm by 500 μm vascular model. Channel, collagen, and Matrigel matrix, 1.5 Pa wall shear stress.	Mechanical stimulation reduced extravasation distance 32.4% and frequency by 53.5%.
Xu [120]	MDA-MB-231 and M624 cells cancer, primary rat brain microvascular endothelial cells (BMECs), primary rat cerebral astrocytes	3D	Circulating signals and cells	Blood–brain barrier model in a chip.	Cancer cell and astrocyte interactions increase brain tumor migration between brain and vascular compartments.
				200 μm by 400 μm, collagen matrix, 0.1 dyne/cm^2^ and 60 μL/h.	
Liu [270]	MCF-7 and MCF-7adm cancer	3D	Treatment	Five parallel gradient-generating networks on a chip using dam and weir structures for cell positioning and seeding to investigate anti-cancer drugs.	GSH levels of two breast cancer cell lines were reduced during arsenic trioxide treatment, resulting in increased chemotherapy sensitivity, and vice versa was found with N-acetyl cystine.
				2 mm by 1 mm by 30 μm, no ECM, 20 μL/h.	
Parekh [275]	MDA-MB-231	Suspended particles	Treatment	Single breast cancer cell selection, drug loading, and fluorescence measurement on a chip.	Cyclosporine A found to greatly increase the cellular uptake of anti-cancer drugs by reducing drug efflux.
				30 mm by 30 mm chip, no ECM, no flow.	
Sarkar [271]	MCF-7	Suspended particles	Treatment	Droplet docking microfluidic microarray to encapsulate single cells to investigate anti-cancer drug influx, efflux, and cytotoxicity.	Confirmation of previous findings and further classification of drug resistance between breast cancer cell lines.
				No dimensions given, no ECM, no flow.	Observed increased drug resistance during the homotypic fusion of cell-sensitive and resistant cell types within droplets.
Zhang [244]	2A4 hybridoma cells	Suspended particles	Treatment	Integrating chip for screening heavy and light chain combinations in antibodies using single-cell trapping, qPCR, and fluorescence to quantify specificity.	Anti-CD45 monoclonal antibodies identified using hybridoma cells.
				50 μm width, no ECM, 180 μL/h.	
Cheng [233]	T47D and BT549 cancer, primary HUVECs	Spheroids	Treatment	Isolated tumor spheroids trapped next to endothelial cell vascular tissue model to analyze nanoparticle drug delivery systems.	The nanoparticle drug Ds-PEG-FA/DOX found to penetrate spheroids of BT549 cancer but not T47D.
				300 μm long, 200 μm wide, 100 μm deep, basement membrane extract matrix, flow rate not specified.	
Qi [273]	MDA-MB-231 and MDA-MB-231/MDR	Subcellular and 2D	Treatment	Antibody-laden pillars for the specific capture of exosomes for isolation.	CD63-laden exosomes isolated at 70% efficiency.
				4 mm channel length, no ECM, 600 μL/h.	Drug content of isolated exosomes found to be significantly different between cell lines and between treatments.
Qiao [257]	A549 Human lung adeno-carcinoma, HEK293T human kidney cell line	Suspended particles	Treatment	Microfluidic encapsulation of oncolytic adenovirus and the BET bromodomain inhibitor JQ1 within PVA microgels for injection into tumors.	Found to extend persistence and accumulation of oncolytic adenovirus within tumors.
				Device dimensions not specified, no ECM, flow rate not specified.	PVA microgels inhibited PD-L1 expression to overcome immune suppression.
Ide [252]	BW5147 (H2k), JCRB9002, and BW5147	Suspended cells	Treatment	Lymph node and T cell–APC interaction model for cell collection and analysis.	Calcium ion flux fluorescent dye in T cells used as metric of activation during serial contact with APCs.
				20 μm well diameter, no ECM, 4200 μL/h.	T cell activation during contact with OVA 257–264 peptide presenting quantified APCs.
Lou [239]	Bone marrow cells	Suspended particles	Treatment	Tunable liposome formation in a microfluidic channel.	Cationic liposomes of 50–750 nm composed of combinations of tailored phospholipid ratios.
				Hydrodynamic focusing cartridges of 10 and 65 µm width, no ECM, 900 mL/h.	Macrophage liposome uptake is modulated by area and volume, and biodistribution in mice showed that <50 nm particles increase clearance rate.
Liu [240]	HeLa	3D	Treatment	Continuous flow co-precipitation of polymers to produce injectable “nanosystems”.	Nanosystem loaded with photosensitive zinc successfully localized to cancer cells to enable photodynamic therapy.
				200 μm wide and 45 μm deep, no ECM, 7200 μL/h.	
Lee [259]	MRC5 fibroblasts, human lung cancer A549 cells	Spheroid	Treatment	Cancer, fibroblast, and endothelial spheroid model for oncolytic virus infection on a chip.	Cancer cells transfected substantially more than bystander cells, which is not observed in 2D cell culture
				500 μm spheroid chambers, collagen I matrix, 0.3 dyne/cm^2^.	HUVEC IFN-B secretion is delayed in 2D compared to the microfluidic model.
Terrell [248]	MDA-MB-231-HER2+ cancer, CTX-TNA2 rat astrocytes	3D	Treatment	Blood–brain barrier model in a chip to investigate monoclonal antibody localization.	Tailored antibody trastuzumab found to localize 3% to healthy brain and 5% to tumor model brain.
				200 μm width, fibronectin matrix, 60 μL/h.	Rate of uptake quantified as 2.7 × 10^3^ to healthy brain and 1.28 × 10^4^ to cancerous brain.
He [238]	Acellular	Suspended particles	Treatment	Hydrodynamic flow for self-assembly of amphiphilic nanoparticles capable of forming a “micelle”.	500 nm to 2 μm giant vesicles formed.
				Channel size not described, no ECM, 5400 μL/h.	

## References

[1] Thomas R.S., Black M.B., Li L., Healy E., Chu T.-M., Bao W., Andersen M.E., Wolfinger R.D. (2012). A Comprehensive Statistical Analysis of Predicting In Vivo Hazard Using High-Throughput In Vitro Screening. Toxicol. Sci..

[2] Selimović Š., Dokmeci M.R., Khademhosseini A. (2013). Organs-on-a-chip for drug discovery. Curr. Opin. Pharmacol..

[3] Frantz C., Stewart K.M., Weaver V.M. (2010). The extracellular matrix at a glance. J. Cell Sci..

[4] Swartz M.A., Fleury M.E. (2007). Interstitial Flow and Its Effects in Soft Tissues. Annu. Rev. Biomed. Eng..

[5] Holen I., Speirs V., Morrissey B., Blyth K. (2017). In vivo models in breast cancer research: Progress, challenges and future directions. Dis. Model. Mech..

[6] Halldorsson S., Lucumi E., Gómez-Sjöberg R., Fleming R.M.T. (2015). Advantages and challenges of microfluidic cell culture in polydimethylsiloxane devices. Biosens. Bioelectron..

[7] Ferlay J., Colombet M., Soerjomataram I., Parkin D.M., Piñeros M., Znaor A., Bray F. (2021). Cancer statistics for the year 2020: An overview. Int. J. Cancer.

[8] Azamjah N., Soltan-Zadeh Y., Zayeri F. (2019). Global Trend of Breast Cancer Mortality Rate: A 25-Year Study. Asian Pac. J. Cancer Prev..

[9] Dong M., Cioffi G., Wang J., Waite K., Ostrom Q., Kruchko C., Lathia J., Rubin J., Berens M., Connor J. (2020). Sex Differences in Cancer Incidence and Survival: A Pan-Cancer Analysis. Cancer Epidemiol. Biomark. Prev..

[10] Franzen N., van Harten W.H., Retèl V.P., Loskill P., van den Eijnden-van Raaij J., IJzerman M. (2019). Impact of organ-on-a-chip technology on pharmaceutical R&D costs. Drug Discov. Today.

[11] Chen W., Dong J., Haiech J., Kilhoffer M.-C., Zeniou M. (2016). Cancer Stem Cell Quiescence and Plasticity as Major Challenges in Cancer Therapy. Stem Cells Int..

[12] Strilic B., Offermanns S. (2017). Intravascular Survival and Extravasation of Tumor Cells. Cancer Cell.

[13] Disibio G., French S.W. (2008). Metastatic Patterns of Cancers: Results From a Large Autopsy Study. Arch. Pathol. Lab. Med..

[14] Soni A., Ren Z., Hameed O., Chanda D., Morgan C.J., Siegal G.P., Wei S. (2015). Breast Cancer Subtypes Predispose the Site of Distant Metastases. Am. J. Clin. Pathol..

[15] Sigdel I., Gupta N., Faizee F., Khare V.M., Tiwari A.K., Tang Y. (2021). Biomimetic Microfluidic Platforms for the Assessment of Breast Cancer Metastasis. Front. Bioeng. Biotechnol..

[16] Krol I., Schwab F.D., Carbone R., Ritter M., Picocci S., De Marni M.L., Stepien G., Franchi G.M., Zanardi A., Rissoglio M.D. (2021). Detection of clustered circulating tumour cells in early breast cancer. Br. J. Cancer.

[17] Schirrmacher V. (2019). From chemotherapy to biological therapy: A review of novel concepts to reduce the side effects of systemic cancer treatment (Review). Int. J. Oncol..

[18] Saeki K., Chang G., Kanaya N., Wu X., Wang J., Bernal L., Ha D., Neuhausen S.L., Chen S. (2021). Mammary cell gene expression atlas links epithelial cell remodeling events to breast carcinogenesis. Commun. Biol..

[19] Cheng Y.-H., Wang C.-H., Hsu K.-F., Lee G.-B. An Integrated Microfluidic Platform for Detecting BRCA1/BRCA2 Gene Mutation and Risk Assessment of Ovarian Cancer. Proceedings of the 2021 21st International Conference on Solid-State Sensors, Actuators and Microsystems (Transducers).

[20] Pradhan S., Smith A.M., Garson C.J., Hassani I., Seeto W.J., Pant K., Arnold R.D., Prabhakarpandian B., Lipke E.A. (2018). A Microvascularized Tumor-mimetic Platform for Assessing Anti-cancer Drug Efficacy. Sci. Rep..

[21] Ayuso J.M., Gillette A., Lugo-Cintrón K., Acevedo-Acevedo S., Gomez I., Morgan M., Heaster T., Wisinski K.B., Palecek S.P., Skala M.C. (2018). Organotypic microfluidic breast cancer model reveals starvation-induced spatial-temporal metabolic adaptations. EBioMedicine.

[22] Funamoto K., Zervantonakis I.K., Liu Y., Ochs C.J., Kim C., Kamm R.D. (2012). A novel microfluidic platform for high-resolution imaging of a three-dimensional cell culture under a controlled hypoxic environment. Lab. Chip.

[23] Tang Y., Soroush F., Sheffield J.B., Wang B., Prabhakarpandian B., Kiani M.F. (2017). A Biomimetic Microfluidic Tumor Microenvironment Platform Mimicking the EPR Effect for Rapid Screening of Drug Delivery Systems. Sci. Rep..

[24] Nashimoto Y., Okada R., Hanada S., Arima Y., Nishiyama K., Miura T., Yokokawa R. (2020). Vascularized cancer on a chip: The effect of perfusion on growth and drug delivery of tumor spheroid. Biomaterials.

[25] Viale G., Regan M.M., Maiorano E., Mastropasqua M.G., Dell’Orto P., Rasmussen B.B., Raffoul J., Neven P., Orosz Z., Braye S. (2007). Prognostic and Predictive Value of Centrally Reviewed Expression of Estrogen and Progesterone Receptors in a Randomized Trial Comparing Letrozole and Tamoxifen Adjuvant Therapy for Postmenopausal Early Breast Cancer: BIG 1-98. J. Clin. Oncol..

[26] Huang M., Wu J., Ling R., Li N. (2020). Quadruple negative breast cancer. Breast Cancer.

[27] Siddharth S., Sharma D. (2018). Racial Disparity and Triple-Negative Breast Cancer in African-American Women: A Multifaceted Affair between Obesity, Biology, and Socioeconomic Determinants. Cancers.

[28] Iwamoto T., Booser D., Valero V., Murray J.L., Koenig K., Esteva F.J., Ueno N.T., Zhang J., Shi W., Qi Y. (2012). Estrogen Receptor (ER) mRNA and ER-Related Gene Expression in Breast Cancers That Are 1% to 10% ER-Positive by Immunohistochemistry. J. Clin. Oncol..

[29] Ross D.S., Zehir A., Brogi E., Konno F., Krystel-Whittemore M., Edelweiss M., Berger M.F., Toy W., Chandarlapaty S., Razavi P. (2019). Immunohistochemical analysis of estrogen receptor in breast cancer with ESR1 mutations detected by hybrid capture-based next-generation sequencing. Mod. Pathol..

[30] Kumar M., Sahu R.K., Goyal A., Sharma S., Kaur N., Mehrotra R., Singh U.R., Hedau S. (2017). BRCA1 Promoter Methylation and Expression-Associations with ER+, PR+ and HER2+ Subtypes of Breast Carcinoma. Asian Pac. J. Cancer Prev..

[31] Verdu M., Trias I., Roman R., Rodon N., Pubill C., Arraiza N., Martinez B., Garcia-Pelaez B., Serrano T., Puig X. (2015). Cross-reactivity of EGFR Mutation-specific Immunohistochemistry Assay in HER2-positive Tumors. Appl. Immunohistochem. Mol. Morphol..

[32] Uliana C.V., Peverari C.R., Afonso A.S., Cominetti M.R., Faria R.C. (2018). Fully disposable microfluidic electrochemical device for detection of estrogen receptor alpha breast cancer biomarker. Biosens. Bioelectron..

[33] Hanahan D., Weinberg R.A. (2011). Hallmarks of Cancer: The Next Generation. Cell.

[34] Hoffmann C., Mao X., Brown-Clay J., Moreau F., Al Absi A., Wurzer H., Sousa B., Schmitt F., Berchem G., Janji B. (2018). Hypoxia promotes breast cancer cell invasion through HIF-1α-mediated up-regulation of the invadopodial actin bundling protein CSRP2. Nat. Sci. Rep..

[35] Marín-Hernández Á., Gallardo-Pérez J.C., Hernández-Reséndiz I., Del Mazo-Monsalvo I., Robledo-Cadena D.X., Moreno-Sánchez R., Rodríguez-Enríquez S. (2017). Hypoglycemia Enhances Epithelial-Mesenchymal Transition and Invasiveness, and Restrains the Warburg Phenotype, in Hypoxic HeLa Cell Cultures and Microspheroids: Hypoglycemia Restrains the Warburg Phenotype in Hypoxic Cancer Cells. J. Cell. Physiol..

[36] Wang Y., Zhou B.P. (2011). Epithelial-mesenchymal transition in breast cancer progression and metastasis. Chin. J. Cancer.

[37] Padmanaban V., Krol I., Suhail Y., Szczerba B.M., Aceto N., Bader J.S., Ewald A.J. (2019). E-cadherin is required for metastasis in multiple models of breast cancer. Nature.

[38] Liu Y.-J., Le Berre M., Lautenschlaeger F., Maiuri P., Callan-Jones A., Heuzé M., Takaki T., Voituriez R., Piel M. (2015). Confinement and Low Adhesion Induce Fast Amoeboid Migration of Slow Mesenchymal Cells. Cell.

[39] Wu J., Jiang J., Chen B., Wang K., Tang Y., Liang X. (2021). Plasticity of cancer cell invasion: Patterns and mechanisms. Transl. Oncol..

[40] Estrella V., Chen T., Lloyd M., Wojtkowiak J., Cornnell H.H., Ibrahim-Hashim A., Bailey K., Balagurunathan Y., Rothberg J.M., Sloane B.F. (2013). Acidity Generated by the Tumor Microenvironment Drives Local Invasion. Cancer Res..

[41] Hanley C.J., Henriet E., Sirka O.K., Thomas G.J., Ewald A.J. (2020). Tumor-Resident Stromal Cells Promote Breast Cancer Invasion through Regulation of the Basal Phenotype. Mol. Cancer Res..

[42] Winkler J., Abisoye-Ogunniyan A., Metcalf K.J., Werb Z. (2020). Concepts of extracellular matrix remodelling in tumour progression and metastasis. Nat. Commun..

[43] Li H., Qiu Z., Li F., Wang C. (2017). The relationship between MMP-2 and MMP-9 expression levels with breast cancer incidence and prognosis. Oncol. Lett..

[44] Moon H., Ospina-Muñoz N., Noe-Kim V., Yang Y., Elzey B.D., Konieczny S.F., Han B. (2020). Subtype-specific characterization of breast cancer invasion using a microfluidic tumor platform. PLoS ONE.

[45] Aw Yong K.M., Ulintz P.J., Caceres S., Cheng X., Bao L., Wu Z., Jiagge E.M., Merajver S.D. (2020). Heterogeneity at the invasion front of triple negative breast cancer cells. Sci. Rep..

[46] Sung K.E., Yang N., Pehlke C., Keely P.J., Eliceiri K.W., Friedl A., Beebe D.J. (2011). Transition to invasion in breast cancer: A microfluidic in vitro model enables examination of spatial and temporal effects. Integr Biol.

[47] Lam S.F., Bishop K.W., Mintz R., Fang L., Achilefu S. (2021). Calcium carbonate nanoparticles stimulate cancer cell reprogramming to suppress tumor growth and invasion in an organ-on-a-chip system. Sci. Rep..

[48] Toh Y.-C., Raja A., Yu H., van Noort D. (2018). A 3D Microfluidic Model to Recapitulate Cancer Cell Migration and Invasion. Bioengineering.

[49] Yankaskas C.L., Thompson K.N., Paul C.D., Vitolo M.I., Mistriotis P., Mahendra A., Bajpai V.K., Shea D.J., Manto K.M., Chai A.C. (2019). A microfluidic assay for the quantification of the metastatic propensity of breast cancer specimens. Nat. Biomed. Eng..

[50] Gioiella F., Urciuolo F., Imparato G., Brancato V., Netti P.A. (2016). An Engineered Breast Cancer Model on a Chip to Replicate ECM-Activation In Vitro during Tumor Progression. Adv. Healthc. Mater..

[51] Mosadegh B., Lockett M.R., Minn K.T., Simon K.A., Gilbert K., Hillier S., Newsome D., Li H., Hall A.B., Boucher D.M. (2015). A paper-based invasion assay: Assessing chemotaxis of cancer cells in gradients of oxygen. Biomaterials.

[52] Mi S., Liu Z., Du Z., Yi X., Sun W. (2019). Three-dimensional microfluidic tumor-macrophage system for breast cancer cell invasion. Biotechnol. Bioeng..

[53] Lugo-Cintrón K.M., Gong M.M., Ayuso J.M., Tomko L.A., Beebe D.J., Virumbrales-Muñoz M., Ponik S.M. (2020). Breast Fibroblasts and ECM Components Modulate Breast Cancer Cell Migration through the Secretion of MMPs in a 3D Microfluidic Co-Culture Model. Cancers.

[54] Liu Z., Han X., Chen R., Zhang K., Li Y., Fruge S., Jang J.h., Ma Y., Qin L. (2017). Microfluidic Mapping of Cancer Cell–Protein Binding Interaction. ACS Appl. Mater. Interfaces.

[55] Han W., Chen S., Yuan W., Fan Q., Tian J., Wang X., Chen L., Zhang X., Wei W., Liu R. (2016). Oriented collagen fibers direct tumor cell intravasation. Proc. Natl. Acad. Sci. USA.

[56] Ginter P.S., Karagiannis G.S., Entenberg D., Lin Y., Condeelis J., Jones J., Oktay M.H. (2019). Tumor Microenvironment of Metastasis (TMEM) Doorways Are Restricted to the Blood Vessel Endothelium in Both Primary Breast Cancers and Their Lymph Node Metastases. Cancers.

[57] Vestweber D., Winderlich M., Cagna G., Nottebaum A.F. (2009). Cell adhesion dynamics at endothelial junctions: VE-cadherin as a major player. Trends Cell Biol..

[58] Sanchez L.R., Borriello L., Entenberg D., Condeelis J.S., Oktay M.H., Karagiannis G.S. (2019). The emerging roles of macrophages in cancer metastasis and response to chemotherapy. J. Leukoc. Biol..

[59] Qian B., Deng Y., Im J.H., Muschel R.J., Zou Y., Li J., Lang R.A., Pollard J.W. (2009). A Distinct Macrophage Population Mediates Metastatic Breast Cancer Cell Extravasation, Establishment and Growth. PLoS ONE.

[60] Linde N., Casanova-Acebes M., Sosa M.S., Mortha A., Rahman A., Farias E., Harper K., Tardio E., Reyes Torres I., Jones J. (2018). Macrophages orchestrate breast cancer early dissemination and metastasis. Nat. Commun..

[61] Oshi M., Tokumaru Y., Asaoka M., Yan L., Satyananda V., Matsuyama R., Matsuhashi N., Futamura M., Ishikawa T., Yoshida K. (2020). M1 Macrophage and M1/M2 ratio defined by transcriptomic signatures resemble only part of their conventional clinical characteristics in breast cancer. Sci. Rep..

[62] Arwert E.N., Harney A.S., Entenberg D., Wang Y., Sahai E., Pollard J.W., Condeelis J.S. (2018). A Unidirectional Transition from Migratory to Perivascular Macrophage Is Required for Tumor Cell Intravasation. Cell Rep..

[63] Munir M.T., Kay M.K., Kang M.H., Rahman M.M., Al-Harrasi A., Choudhury M., Moustaid-Moussa N., Hussain F., Rahman S.M. (2021). Tumor-Associated Macrophages as Multifaceted Regulators of Breast Tumor Growth. Int. J. Mol. Sci..

[64] Sainson R.C.A., Johnston D.A., Chu H.C., Holderfield M.T., Nakatsu M.N., Crampton S.P., Davis J., Conn E., Hughes C.C.W. (2008). TNF primes endothelial cells for angiogenic sprouting by inducing a tip cell phenotype. Blood.

[65] (2011). Yvette Drabsch & Peter ten Dijk TGF-β Signaling in Breast Cancer Cell Invasion and Bone Metastasis. J Mammary Gland Biol Neoplasia.

[66] Rodriguez-Vita J., Fischer A. (2017). Notch signaling facilitates crossing of endothelial barriers by tumor cells. Mol. Cell. Oncol..

[67] Lopes-Bastos B.M., Jiang W.G., Cai J. (2016). Tumour–Endothelial Cell Communications: Important and Indispensable Mediators of Tumour Angiogenesis. Anticancer Res..

[68] Di Modica M., Regondi V., Sandri M., Iorio M.V., Zanetti A., Tagliabue E., Casalini P., Triulzi T. (2017). Breast cancer-secreted miR-939 downregulates VE-cadherin and destroys the barrier function of endothelial monolayers. Cancer Lett..

[69] Kanchan R.K., Siddiqui J.A., Mahapatra S., Batra S.K., Nasser M.W. (2020). microRNAs Orchestrate Pathophysiology of Breast Cancer Brain Metastasis: Advances in Therapy. Mol. Cancer.

[70] Balzer E.M., Tong Z., Paul C.D., Hung W., Stroka K.M., Boggs A.E., Martin S.S., Konstantopoulos K. (2012). Physical confinement alters tumor cell adhesion and migration phenotypes. FASEB J..

[71] Zervantonakis I.K., Hughes-Alford S.K., Charest J.L., Condeelis J.S., Gertler F.B., Kamm R.D. (2012). Three-dimensional microfluidic model for tumor cell intravasation and endothelial barrier function. Proc. Natl. Acad. Sci. USA.

[72] Buchanan C.F., Voigt E.E., Szot C.S., Freeman J.W., Vlachos P.P., Rylander M.N. (2014). Three-Dimensional Microfluidic Collagen Hydrogels for Investigating Flow-Mediated Tumor-Endothelial Signaling and Vascular Organization. Tissue Eng. Part C Methods.

[73] Choi Y., Hyun E., Seo J., Blundell C., Kim H.C., Lee E., Lee S.H., Moon A., Moon W.K., Huh D. (2015). A microengineered pathophysiological model of early-stage breast cancer. Lab. Chip.

[74] Mi S., Du Z., Xu Y., Wu Z., Qian X., Zhang M., Sun W. (2016). Microfluidic co-culture system for cancer migratory analysis and anti-metastatic drugs screening. Sci. Rep..

[75] Cui X., Guo W., Sun Y., Sun B., Hu S., Sun D., Lam R.H.W. (2017). A microfluidic device for isolation and characterization of transendothelial migrating cancer cells. Biomicrofluidics.

[76] Nagaraju S., Truong D., Mouneimne G., Nikkhah M. (2018). Microfluidic Tumor–Vascular Model to Study Breast Cancer Cell Invasion and Intravasation. Adv. Healthc. Mater..

[77] Shirure V.S., Bi Y., Curtis M.B., Lezia A., Goedegebuure M.M., Goedegebuure S.P., Aft R., Fields R.C., George S.C. (2018). Tumor-on-a-chip platform to investigate progression and drug sensitivity in cell lines and patient-derived organoids. Lab. Chip.

[78] Cho H.-Y., Choi J.-H., Kim K.-J., Shin M., Choi J.-W. (2021). Microfluidic System to Analyze the Effects of Interleukin 6 on Lymphatic Breast Cancer Metastasis. Front. Bioeng. Biotechnol..

[79] Leone K., Poggiana C., Zamarchi R. (2018). The Interplay between Circulating Tumor Cells and the Immune System: From Immune Escape to Cancer Immunotherapy. Diagnostics.

[80] Zhao H., Wang J., Kong X., Li E., Liu Y., Du X., Kang Z., Tang Y., Kuang Y., Yang Z. (2016). CD47 Promotes Tumor Invasion and Metastasis in Non-small Cell Lung Cancer. Sci. Rep..

[81] Wang X., Sun Q., Liu Q., Wang C., Yao R., Wang Y. (2016). CTC immune escape mediated by PD-L1. Med. Hypotheses.

[82] Twomey J., Zhang B. (2019). Circulating Tumor Cells Develop Resistance to TRAIL-Induced Apoptosis Through Autophagic Removal of Death Receptor 5: Evidence from an In Vitro Model. Cancers.

[83] Han H., Sung J.Y., Kim S.-H., Yun U.-J., Kim H., Jang E.-J., Yoo H.-E., Hong E.K., Goh S.-H., Moon A. (2021). Fibronectin regulates anoikis resistance via cell aggregate formation. Cancer Lett..

[84] Mitchell M.J., King M.R. (2013). Fluid shear stress sensitizes cancer cells to receptor-mediated apoptosis via trimeric death receptors. New J. Phys..

[85] Ward M.P., Kane E.L., Norris A.L., Mohamed B.M., Kelly T., Bates M., Clarke A., Brady N., Martin C.M., Brooks R.D. (2021). Platelets, immune cells and the coagulation cascade; friend or foe of the circulating tumour cell?. Mol. Cancer.

[86] Liu X., Taftaf R., Kawaguchi M., Chang Y.-F., Chen W., Entenberg D., Zhang Y., Gerratana L., Huang S., Patel D.B. (2019). Homophilic CD44 Interactions Mediate Tumor Cell Aggregation and Polyclonal Metastasis in Patient-Derived Breast Cancer Models. Cancer Discov..

[87] Szczerba B.M., Castro-Giner F., Vetter M., Krol I., Gkountela S., Landin J., Scheidmann M.C., Donato C., Scherrer R., Singer J. (2019). Neutrophils escort circulating tumour cells to enable cell cycle progression. Nature.

[88] Hess K.R., Varadhachary G.R., Taylor S.H., Wei W., Raber M.N., Lenzi R., Abbruzzese J.L. (2006). Metastatic patterns in adenocarcinoma. Cancer.

[89] Wong G.L., Abu Jalboush S., Lo H.-W. (2020). Exosomal MicroRNAs and Organotropism in Breast Cancer Metastasis. Cancers.

[90] Wu Q., Li J., Zhu S., Wu J., Chen C., Liu Q., Wei W., Zhang Y., Sun S. (2017). Breast cancer subtypes predict the preferential site of distant metastases: A SEER based study. Oncotarget.

[91] Yu T., Wang C., Xie M., Zhu C., Shu Y., Tang J., Guan X. (2021). Heterogeneity of CTC contributes to the organotropism of breast cancer. Biomed. Pharmacother..

[92] Padua D., Zhang X.H.-F., Wang Q., Nadal C., Gerald W.L., Gomis R.R., Massagué J. (2008). TGFβ Primes Breast Tumors for Lung Metastasis Seeding through Angiopoietin-like 4. Cell.

[93] Zhang X.H.-F., Wang Q., Gerald W., Hudis C.A., Norton L., Smid M., Foekens J.A., Massagué J. (2009). Latent Bone Metastasis in Breast Cancer Tied to Src-Dependent Survival Signals. Cancer Cell.

[94] Yoneda T., Williams P.J., Hiraga T., Niewolna M., Nishimura R. (2001). A Bone-Seeking Clone Exhibits Different Biological Properties from the MDA-MB-231 Parental Human Breast Cancer Cells and a Brain-Seeking Clone In Vivo and In Vitro. J. Bone Miner. Res..

[95] Hoshino A., Costa-Silva B., Shen T.-L., Rodrigues G., Hashimoto A., Tesic Mark M., Molina H., Kohsaka S., Di Giannatale A., Ceder S. (2015). Tumour exosome integrins determine organotropic metastasis. Nature.

[96] Osmani N., Follain G., García León M.J., Lefebvre O., Busnelli I., Larnicol A., Harlepp S., Goetz J.G. (2019). Metastatic Tumor Cells Exploit Their Adhesion Repertoire to Counteract Shear Forces during Intravascular Arrest. Cell Rep..

[97] Yadavalli S., Jayaram S., Manda S.S., Madugundu A.K., Nayakanti D.S., Tan T.Z., Bhat R., Rangarajan A., Chatterjee A., Gowda H. (2017). Data-Driven Discovery of Extravasation Pathway in Circulating Tumor Cells. Sci. Rep..

[98] (2016). Sang A Park and Young-Min Hyuncorresponding author Neutrophil Extravasation Cascade: What Can We Learn from Two-photon Intravital Imaging?. Immune Netw..

[99] Yang J., Nie J., Ma X., Wei Y., Peng Y., Wei X. (2019). Targeting PI3K in cancer: Mechanisms and advances in clinical trials. Mol. Cancer.

[100] Zhang X.H.-F., Jin X., Malladi S., Zou Y., Wen Y.H., Brogi E., Smid M., Foekens J.A., Massagué J. (2013). Selection of Bone Metastasis Seeds by Mesenchymal Signals in the Primary Tumor Stroma. Cell.

[101] Hosaka K., Yang Y., Seki T., Fischer C., Dubey O., Fredlund E., Hartman J., Religa P., Morikawa H., Ishii Y. (2016). Pericyte–fibroblast transition promotes tumor growth and metastasis. Proc. Natl. Acad. Sci. USA.

[102] Lugassy C., Kleinman H., Barnhill R. (2020). Pericyte mimicry: An embryogenesis-derived program of extravascular tumor cell migration. Tumor Vascularization.

[103] Hurtado P., Martínez-Pena I., Piñeiro R. (2020). Dangerous Liaisons: Circulating Tumor Cells (CTCs) and Cancer-Associated Fibroblasts (CAFs). Cancers.

[104] Zeng Q., Li W., Lu D., Wu Z., Duan H., Luo Y., Feng J., Yang D., Fu L., Yan X. (2012). CD146, an epithelial-mesenchymal transition inducer, is associated with triple-negative breast cancer. Proc. Natl. Acad. Sci. USA.

[105] Mostert B., Kraan J., Bolt-de Vries J., van der Spoel P., Sieuwerts A.M., Schutte M., Timmermans A.M., Foekens R., Martens J.W.M., Gratama J.-W. (2011). Detection of circulating tumor cells in breast cancer may improve through enrichment with anti-CD146. Breast Cancer Res. Treat..

[106] Imbert A.-M., Garulli C., Choquet E., Koubi M., Aurrand-Lions M., Chabannon C. (2012). CD146 Expression in Human Breast Cancer Cell Lines Induces Phenotypic and Functional Changes Observed in Epithelial to Mesenchymal Transition. PLoS ONE.

[107] Mayo V., Bowles A., Wubker L., Ortiz I., Cordoves A., Cote R., Correa D., Agarwal A. (2021). Human-derived osteoblast-like cells and pericyte-like cells induce distinct metastatic phenotypes in primary breast cancer cells. Exp. Biol. Med..

[108] Zhang D., LaFortune T.A., Krishnamurthy S., Esteva F.J., Cristofanilli M., Liu P., Lucci A., Singh B., Hung M.-C., Hortobagyi G.N. (2009). Epidermal Growth Factor Receptor Tyrosine Kinase Inhibitor Reverses Mesenchymal to Epithelial Phenotype and Inhibits Metastasis in Inflammatory Breast Cancer. Clin. Cancer Res..

[109] Huang P., Chen A., He W., Li Z., Zhang G., Liu Z., Liu G., Liu X., He S., Xiao G. (2017). BMP-2 induces EMT and breast cancer stemness through Rb and CD44. Cell Death Discov..

[110] Armbrecht L., Rutschmann O., Szczerba B.M., Nikoloff J., Aceto N., Dittrich P.S. (2020). Quantification of Protein Secretion from Circulating Tumor Cells in Microfluidic Chambers. Adv. Sci..

[111] Park J., Park S., Hyun K.A., Jung H.-I. (2021). Microfluidic recapitulation of circulating tumor cell–neutrophil clusters *via* double spiral channel-induced deterministic encapsulation. Lab. Chip.

[112] Wheeler S.E., Clark A.M., Taylor D.P., Young C.L., Pillai V.C., Stolz D.B., Venkataramanan R., Lauffenburger D., Griffith L., Wells A. (2014). Spontaneous dormancy of metastatic breast cancer cells in an all human liver microphysiologic system. Br. J. Cancer.

[113] Riahi R., Yang Y.L., Kim H., Jiang L., Wong P.K., Zohar Y. (2014). A microfluidic model for organ-specific extravasation of circulating tumor cells. Biomicrofluidics.

[114] Aleman J., Skardal A. (2019). A multi-site metastasis-on-a-chip microphysiological system for assessing metastatic preference of cancer cells: ALEMAN AND SKARDAL. Biotechnol. Bioeng..

[115] Song J., Miermont A., Lim C.T., Kamm R.D. (2018). A 3D microvascular network model to study the impact of hypoxia on the extravasation potential of breast cell lines. Sci. Rep..

[116] Marturano-Kruik A., Nava M.M., Yeager K., Chramiec A., Hao L., Robinson S., Guo E., Raimondi M.T., Vunjak-Novakovic G. (2018). Human bone perivascular niche-on-a-chip for studying metastatic colonization. Proc. Natl. Acad. Sci. USA.

[117] Jeon J.S., Bersini S., Gilardi M., Dubini G., Charest J.L., Moretti M., Kamm R.D. (2015). Human 3D vascularized organotypic microfluidic assays to study breast cancer cell extravasation. Proc. Natl. Acad. Sci. USA.

[118] Bersini S., Jeon J.S., Dubini G., Arrigoni C., Chung S., Charest J.L., Moretti M., Kamm R.D. (2014). A microfluidic 3D in vitro model for specificity of breast cancer metastasis to bone. Biomaterials.

[119] Mei X., Middleton K., Shim D., Wan Q., Xu L., Ma Y.-H.V., Devadas D., Walji N., Wang L., Young E.W.K. (2019). Microfluidic platform for studying osteocyte mechanoregulation of breast cancer bone metastasis. Integr. Biol..

[120] Xu H., Li Z., Yu Y., Sizdahkhani S., Ho W.S., Yin F., Wang L., Zhu G., Zhang M., Jiang L. (2016). A dynamic in vivo-like organotypic blood-brain barrier model to probe metastatic brain tumors. Sci. Rep..

[121] Wang L. (2017). Early Diagnosis of Breast Cancer. Sensors.

[122] Houssami N., Ciatto S., Martinelli F., Bonardi R., Duffy S.W. (2009). Early detection of second breast cancers improves prognosis in breast cancer survivors. Ann. Oncol..

[123] Jaglan P., Dass R., Duhan M. (2019). Breast Cancer Detection Techniques: Issues and Challenges. J. Inst. Eng. India Ser. B.

[124] Ignatiadis M., Sledge G.W., Jeffrey S.S. (2021). Liquid biopsy enters the clinic—implementation issues and future challenges. Nat. Rev. Clin. Oncol..

[125] Alimirzaie S., Bagherzadeh M., Akbari M.R. (2019). Liquid biopsy in breast cancer: A comprehensive review. Clin. Genet..

[126] Akgönüllü S., Bakhshpour M., Pişkin A.K., Denizli A. (2021). Microfluidic Systems for Cancer Diagnosis and Applications. Micromachines.

[127] Guo B., Oliver T.G. (2019). Partners in Crime: Neutrophil–CTC Collusion in Metastasis. Trends Immunol..

[128] Kulasinghe A., Zhou J., Kenny L., Papautsky I., Punyadeera C. (2019). Capture of Circulating Tumour Cell Clusters Using Straight Microfluidic Chips. Cancers.

[129] Duoma S., Van Laar T., Zevenhoven J., Meuwissen R., Van Garderen E., Peeper D.S. (2004). Suppression of anoikis and induction of metastasis by the neurotrophic receptor TRKB. Nature.

[130] Sachdev D., Zhang X., Matise I., Gaillard-Kelly M., Yee D. (2010). The type I insulin-like growth factor receptor regulates cancer metastasis independently of primary tumor growth by promoting invasion and survival. Oncogene.

[131] Ghadially H., Brown L., Lloyd C., Lewis L., Lewis A., Dillon J., Sainson R., Jovanovic J., Tigue N.J., Bannister D. (2017). MHC class I chain-related protein A and B (MICA and MICB) are predominantly expressed intracellularly in tumour and normal tissue. Br. J. Cancer.

[132] Chen Q., Zhang X.H.-F., Massagué J. (2011). Macrophage Binding to Receptor VCAM-1 Transmits Survival Signals in Breast Cancer Cells that Invade the Lungs. Cancer Cell.

[133] Bernson E., Christenson K., Pesce S., Pasanen M., Marcenaro E., Sivori S., Thorén F.B. (2019). Downregulation of HLA Class I Renders Inflammatory Neutrophils More Susceptible to NK Cell-Induced Apoptosis. Front. Immunol..

[134] Schmiedel D., Mandelboim O. (2018). NKG2D Ligands–Critical Targets for Cancer Immune Escape and Therapy. Front. Immunol..

[135] Mammadova-Bach E., Mangin P., Lanza F., Gachet C. (2015). Platelet integrin Alpha6beta1 controls lung metastasis through direct binding to cell-derived ADAM9. JCI Insight.

[136] Takagi S., Sato S., Oh-hara T., Takami M., Koike S., Mishima Y., Hatake K., Fujita N. (2013). Platelets promote tumor growth and metastasis via direct interaction between Aggrus/podoplanin and CLEC-2. PLoS ONE.

[137] Gil-Bernabe A.M., Ferjancic S., Tlalka M., Zhao L., Allen P.D., Im J.H., Watson K., Hill S.A., Amirkhosravi A., Francis J.L. (2012). Recruitment of monocytes/macrophages by tissue factor-mediated coagulation is essential for metastatic cell survival and premetastatic niche establishment in mice. Blood.

[138] Furlow P.W., Xhang S., Soong T.D., Halberg N., Goodarzi H., Mangrum C., Wum Y.G., Elemento O., Tavazoie S.F. (2015). Mechanosensitive Pannexin-1 channels mediate microvascular metastatic cell survival. Nat. Cell Biol..

[139] Vora D.H.H., Patel N.A., Rajvik K.N., Mehta S.V., Brahmbhatt B.V., Shah M.J., Shukla S.N., Shah P.M. (2009). Cytokeratin and Vimentin Expression in Breast Cancer. Int. J. Biol. Markers.

[140] Gradilone A., Raimondi C., Nicolazzo C., Petracca A., Gandini O., Vincenzi B., Naso G., Aglianò A.M., Cortesi E., Gazzaniga P. (2011). Circulating tumour cells lacking cytokeratin in breast cancer: The importance of being mesenchymal. J. Cell. Mol. Med..

[141] Karimi N., Oloomi M., Orafa Z. (2020). Circulating Tumor Cells Detection in Patients with Early Breast Cancer Using MACS Immunomagnetic Flow Cytometry. Avicenna J. Med. Biotechnol..

[142] Tarhan M.O., Gonel A., Kucukzeybek Y., Erten C., Cuhadar S., Yigit S.C., Atay A., Somali I., Dirican A., Demir L. (2013). Prognostic Significance of Circulating Tumor Cells and Serum CA15-3 Levels in Metastatic Breast Cancer, Single Center Experience, Preliminary Results. Asian Pac. J. Cancer Prev..

[143] Charbonneau H., Tonks N.K., Walsh K.A., Fischer E.H. (1988). The leukocyte common antigen (CD45): A putative receptor-linked protein tyrosine phosphatase. Proc. Natl. Acad. Sci. USA.

[144] Peeters D.J.E., Van den Eynden G.G., van Dam P.-J., Prové A., Benoy I.H., van Dam P.A., Vermeulen P.B., Pauwels P., Peeters M., Van Laere S.J. (2011). Circulating tumour cells in the central and the peripheral venous compartment in patients with metastatic breast cancer. Br. J. Cancer.

[145] Mendelaar P.A.J., Kraan J., Van M., Zeune L.L., Terstappen L.W.M.M., Oomen-de Hoop E., Martens J.W.M., Sleijfer S. (2021). Defining the dimensions of circulating tumor cells in a large series of breast, prostate, colon, and bladder cancer patients. Mol. Oncol..

[146] Jiang X., Wong K.H.K., Khankhel A.H., Zeinali M., Reategui E., Phillips M.J., Luo X., Aceto N., Fachin F., Hoang A.N. (2017). Microfluidic isolation of platelet-covered circulating tumor cells. Lab. Chip.

[147] Au S.H., Edd J., Stoddard A.E., Wong K.H.K., Fachin F., Maheswaran S., Haber D.A., Stott S.L., Kapur R., Toner M. (2017). Microfluidic isolation of circulating tumor cell clusters by size and asymmetry. Sci. Rep..

[148] Gertler R., Rosenberg R., Fuehrer K., Dahm M., Nekarda H., Siewert J.R., Allgayer H., Heiss M.M., Schildberg F.W. (2003). Detection of Circulating Tumor Cells in Blood Using an Optimized Density Gradient Centrifugation. Molecular Staging of Cancer.

[149] Deliorman M., Janahi F.K., Sukumar P., Glia A., Alnemari R., Fadl S., Chen W., Qasaimeh M.A. (2020). AFM-compatible microfluidic platform for affinity-based capture and nanomechanical characterization of circulating tumor cells. Microsyst. Nanoeng..

[150] Alexandrova A., Antonova N., Marina S., Shamray E.A., Cherkashina O.V. (2017). Evaluation of the elastic properties and topography of leukocytes’ surface in patients with type 2 diabetes mellitus using atomic force microscope. Ser. Biomech..

[151] Zhou Z.L., Hui T.H., Tang B., Ngan A.H.W. (2014). Accurate measurement of stiffness of leukemia cells and leukocytes using an optical trap by a rate-jump method. RSC Adv..

[152] Han S.-I., Joo Y.-D., Han K.-H. (2013). An electrorotation technique for measuring the dielectric properties of cells with simultaneous use of negative quadrupolar dielectrophoresis and electrorotation. The Analyst.

[153] Cristofanilli M., Broglio K.R., Guarneri V., Jackson S., Fritsche H.A., Islam R., Dawood S., Reuben J.M., Kau S.-W., Lara J.M. (2007). Circulating Tumor Cells in Metastatic Breast Cancer: Biologic Staging Beyond Tumor Burden. Clin. Breast Cancer.

[154] Cristofanilli M., Stopeck A., Reuben J.M. (2004). Circulating Tumor Cells, Disease Progression, and Survival in Metastatic Breast Cancer. N. Engl. J. Med..

[155] Nagrath S., Sequist L.V., Maheswaran S., Bell D.W., Irimia D., Ulkus L., Smith M.R., Kwak E.L., Digumarthy S., Muzikansky A. (2007). Isolation of rare circulating tumour cells in cancer patients by microchip technology. Nature.

[156] Bankó P., Lee S.Y., Nagygyörgy V., Zrínyi M., Chae C.H., Cho D.H., Telekes A. (2019). Technologies for circulating tumor cell separation from whole blood. J. Hematol. Oncol..

[157] Mohamed H., Murray M., Turner J.N., Caggana M. (2009). Isolation of tumor cells using size and deformation. J. Chromatogr. A.

[158] Sarioglu A.F., Aceto N., Kojic N., Donaldson M.C., Zeinali M., Hamza B., Engstrom A., Zhu H., Sundaresan T.K., Miyamoto D.T. (2015). A microfluidic device for label-free, physical capture of circulating tumor cell clusters. Nat. Methods.

[159] Marrella A., Fedi A., Varani G., Vaccari I., Fato M., Firpo G., Guida P., Aceto N., Scaglione S. (2021). High blood flow shear stress values are associated with circulating tumor cells cluster disaggregation in a multi-channel microfluidic device. PLoS ONE.

[160] Regmi S., Fu A., Luo K.Q. (2017). High Shear Stresses under Exercise Condition Destroy Circulating Tumor Cells in a Microfluidic System. Sci. Rep..

[161] Luo H., Zhao C., Song K., Liu D., Ma W., Yu X., Su H., Zhang Z., Zohar Y., Lee Y.-K. (2019). A nonlinear two-degree-of-freedom mass–damper–spring model to predict the isolation of circulating tumor cells in microfluidic-elasto-filtration devices. Microfluid. Nanofluidics.

[162] Gascoyne P., Shim S. (2014). Isolation of Circulating Tumor Cells by Dielectrophoresis. Cancers.

[163] de Oliveira R.A.G., Nicoliche C.Y.N., Pasqualeti A.M., Shimizu F.M., Ribeiro I.R., Melendez M.E., Carvalho A.L., Gobbi A.L., Faria R.C., Lima R.S. (2018). Low-Cost and Rapid-Production Microfluidic Electrochemical Double-Layer Capacitors for Fast and Sensitive Breast Cancer Diagnosis. Anal. Chem..

[164] Plouffe B.D., Murthy S.K., Lewis L.H. (2015). Fundamentals and application of magnetic particles in cell isolation and enrichment: A review. Rep. Prog. Phys..

[165] Wyatt Shields IV C., Reyes C.D., López G.P. (2015). Microfluidic cell sorting: A review of the advances in the separation of cells from debulking to rare cell isolation. Lab. Chip.

[166] Atajanov A., Zhbanov A., Yang S. (2018). Sorting and manipulation of biological cells and the prospects for using optical forces. Micro Nano Syst. Lett..

[167] Hu X., Zhu D., Chen M., Chen K., Liu H., Liu W., Yang Y. (2019). Precise and non-invasive circulating tumor cell isolation based on optical force using homologous erythrocyte binding. Lab. Chip.

[168] Zheng X., Jiang L., Schroeder J., Stopeck A., Zohar Y. (2014). Isolation of viable cancer cells in antibody-functionalized microfluidic devices. Biomicrofluidics.

[169] Zheng X., Cheung L.S.-L., Schroeder J.A., Jiang L., Zohar Y. (2011). A high-performance microsystem for isolating circulating tumor cells. Lab. Chip.

[170] Yap Y.-S., Leong M.C., Chua Y.W., Loh K.W.J., Lee G.E., Lim E.H., Dent R., Ng R.C.H., Lim J.H.-C., Singh G. (2019). Detection and prognostic relevance of circulating tumour cells (CTCs) in Asian breast cancers using a label-free microfluidic platform. PLoS ONE.

[171] Murlidhar V., Rivera-Báez L., Nagrath S. (2016). Affinity Versus Label-Free Isolation of Circulating Tumor Cells: Who Wins?. Small.

[172] Lakshmi S., Hughes T.A., Priya S. (2021). Exosomes and exosomal RNAs in breast cancer: A status update. Eur. J. Cancer.

[173] Malla R.R., Pandrangi S., Kumari S., Gavara M.M., Badana A.K. (2018). Exosomal tetraspanins as regulators of cancer progression and metastasis and novel diagnostic markers. Asia Pac. J. Clin. Oncol..

[174] Tomiyama T., Yang G.-X., Zhao M., Zhang W., Tanaka H., Wang J., Leung P.S., Okazaki K., He X.-S., Lu Q. (2017). The modulation of co-stimulatory molecules by circulating exosomes in primary biliary cirrhosis. Cell. Mol. Immunol..

[175] Lynch S., Santos S.G., Campbell E.C., Nimmo A.M.S., Botting C., Prescott A., Antoniou A.N., Powis S.J. (2009). Novel MHC Class I Structures on Exosomes. J. Immunol..

[176] Jella K.K., Yu L., Yue Q., Friedman D., Duke B.J., Alli A.A. (2016). Exosomal GAPDH from Proximal Tubule Cells Regulate ENaC Activity. PLoS ONE.

[177] Thakur B.K., Zhang H., Becker A., Matei I., Huang Y., Costa-Silva B., Zheng Y., Hoshino A., Brazier H., Xiang J. (2014). Double-stranded DNA in exosomes: A novel biomarker in cancer detection. Cell Res..

[178] Sun Z., Shi K., Yang S., Liu J., Zhou Q., Wang G., Song J., Li Z., Zhang Z., Yuan W. (2018). Effect of exosomal miRNA on cancer biology and clinical applications. Mol. Cancer.

[179] Xie Y., Dang W., Zhang S., Yue W., Yang L., Zhai X., Yan Q., Lu J. (2019). The role of exosomal noncoding RNAs in cancer. Mol. Cancer.

[180] Yamashita T., Kamada H., Kanasaki S., Maeda Y., Nagano K., Abe Y., Inoue M., Yoshioka Y., Tsutsumi Y., Katayama S. (2013). Epidermal growth factor receptor localized to exosome membranes as a possible biomarker for lung cancer diagnosis. Pharmazie.

[181] Chairoungdua A., Smith D., Pochard P., Hull M., Caplan M. (2010). Exosome release of B-catenin: A novel mechanism that antagonizes Wnt signaling. J. Cell Biol..

[182] Krawczyk M.A., Pospieszynska A., Styczewska M., Bien E., Sawicki S., Marino Gammazza A., Fucarino A., Gorska-Ponikowska M. (2020). Extracellular Chaperones as Novel Biomarkers of Overall Cancer Progression and Efficacy of Anticancer Therapy. Appl. Sci..

[183] Olejarz W., Dominiak A. (2020). Tumor-Derived Exosomes in Immunosuppression and Immunotherapy. J. Immunol. Res..

[184] Dong X., Bai X., Ni J., Zhang H., Duan W., Graham P., Li Y. (2020). Exosomes and breast cancer drug resistance. Cell Death Dis..

[185] Huang J., Ding Z., Luo Q., Xu W. (2019). Cancer cell-derived exosomes promote cell proliferation and inhibit cell apoptosis of both normal lung fibroblasts and non-small cell lung cancer cell through delivering alpha-smooth muscle actin. Am. J. Transl. Res..

[186] Tang Q., Cheng J., Cao X., Surowy H., Burwinkel B. (2016). Blood-based DNA methylation as biomarker for breast cancer: A systematic review. Clin. Epigenetics.

[187] Li G., Tang W., Yang F. (2020). Cancer Liquid Biopsy Using Integrated Microfluidic Exosome Analysis Platforms. Biotechnol. J..

[188] Vaidyanathan R., Naghibosadat M., Rauf S., Korbie D., Carrascosa L.G., Shiddiky M.J.A., Trau M. (2014). Detecting Exosomes Specifically: A Multiplexed Device Based on Alternating Current Electrohydrodynamic Induced *Nanoshearing*. Anal. Chem..

[189] Sina A.A.I., Vaidyanathan R., Dey S., Carrascosa L.G., Shiddiky M.J.A., Trau M. (2016). Real time and label free profiling of clinically relevant exosomes. Sci. Rep..

[190] Fang S., Tian H., Li X., Jin D., Li X., Kong J., Yang C., Yang X., Lu Y., Luo Y. (2017). Clinical application of a microfluidic chip for immunocapture and quantification of circulating exosomes to assist breast cancer diagnosis and molecular classification. PLoS ONE.

[191] Dong X., Chi J., Zheng L., Ma B., Li Z., Wang S., Zhao C., Liu H. (2019). Efficient isolation and sensitive quantification of extracellular vesicles based on an integrated ExoID-Chip using photonic crystals. Lab. Chip.

[192] Gao Y., Qiang L., Chu Y., Han Y., Zhang Y., Han L. (2020). Microfluidic chip for multiple detection of miRNA biomarkers in breast cancer based on three-segment hybridization. AIP Adv..

[193] Terzi M.Y., Izmirli M., Gogebakan B. (2016). The cell fate: Senescence or quiescence. Mol. Biol. Rep..

[194] Coller H.A., Sang L., Roberts J.M. (2006). A New Description of Cellular Quiescence. PLoS Biol..

[195] Wilson A., Laurenti E., Oser G., van der Wath R.C., Blanco-Bose W., Jaworski M., Offner S., Dunant C.F., Eshkind L., Bockamp E. (2008). Hematopoietic Stem Cells Reversibly Switch from Dormancy to Self-Renewal during Homeostasis and Repair. Cell.

[196] Li L., Clevers H. (2010). Coexistence of Quiescent and Active Adult Stem Cells in Mammals. Science.

[197] Wang X., Fujimaki K., Mitchell G.C., Kwon J.S., Della Croce K., Langsdorf C., Zhang H.H., Yao G. (2017). Exit from quiescence displays a memory of cell growth and division. Nat. Commun..

[198] Kwon J.S., Everetts N.J., Wang X., Wang W., Della Croce K., Xing J., Yao G. (2017). Controlling Depth of Cellular Quiescence by an Rb-E2F Network Switch. Cell Rep..

[199] Llorens-Bobadilla E., Zhao S., Baser A., Saiz-Castro G., Zwadlo K., Martin-Villalba A. (2015). Single-Cell Transcriptomics Reveals a Population of Dormant Neural Stem Cells that Become Activated upon Brain Injury. Cell Stem Cell.

[200] Rodgers J.T., King K.Y., Brett J.O., Cromie M.J., Charville G.W., Maguire K.K., Brunson C., Mastey N., Liu L., Tsai C.-R. (2014). mTORC1 controls the adaptive transition of quiescent stem cells from G0 to GAlert. Nature.

[201] Orford K.W., Scadden D.T. (2008). Deconstructing stem cell self-renewal: Genetic insights into cell-cycle regulation. Nat. Rev. Genet..

[202] Sampieri K., Fodde R. (2012). Cancer stem cells and metastasis. Semin. Cancer Biol..

[203] Zhou J., Wulfkuhle J., Zhang H., Gu P., Yang Y., Deng J., Margolick J.B., Liotta L.A., Petricoin E., Zhang Y. (2007). Activation of the PTEN/mTOR/STAT3 pathway in breast cancer stem-like cells is required for viability and maintenance. Proc. Natl. Acad. Sci. USA.

[204] Lim P.K., Bliss S.A., Patel S.A., Taborga M., Dave M.A., Gregory L.A., Greco S.J., Bryan M., Patel P.S., Rameshwar P. (2011). Gap Junction–Mediated Import of MicroRNA from Bone Marrow Stromal Cells Can Elicit Cell Cycle Quiescence in Breast Cancer Cells. Cancer Res..

[205] Li S., Kennedy M., Payne S., Kennedy K., Seewaldt V.L., Pizzo S.V., Bachelder R.E. (2014). Model of Tumor Dormancy/Recurrence after Short-Term Chemotherapy. PLoS ONE.

[206] Ono M., Kosaka N., Tominaga N., Yoshioka Y., Takeshita F., Takahashi R., Yoshida M., Tsuda H., Tamura K., Ochiya T. (2014). Exosomes from bone marrow mesenchymal stem cells contain a microRNA that promotes dormancy in metastatic breast cancer cells. Sci. Signal..

[207] Bliss S.A., Sinha G., Sandiford O.A., Williams L.M., Engelberth D.J., Guiro K., Isenalumhe L.L., Greco S.J., Ayer S., Bryan M. (2016). Mesenchymal Stem Cell–Derived Exosomes Stimulate Cycling Quiescence and Early Breast Cancer Dormancy in Bone Marrow. Cancer Res..

[208] Lee S.H., Reed-Newman T., Anant S., Ramasamy T.S. (2020). Regulatory Role of Quiescence in the Biological Function of Cancer Stem Cells. Stem Cell Rev. Rep..

[209] Faley S.L., Copland M., Wlodkowic D., Kolch W., Seale K.T., Wikswo J.P., Cooper J.M. (2009). Microfluidic single cell arrays to interrogate signalling dynamics of individual, patient-derived hematopoietic stem cells. Lab. Chip.

[210] Lecault V., VanInsberghe M., Sekulovic S., Knapp D.J.H.F., Wohrer S., Bowden W., Viel F., McLaughlin T., Jarandehei A., Miller M. (2011). High-throughput analysis of single hematopoietic stem cell proliferation in microfluidic cell culture arrays. Nat. Methods.

[211] Zhang W., Kai K., Choi D.S., Iwamoto T., Nguyen Y.H., Wong H., Landis M.D., Ueno N.T., Chang J., Qin L. (2012). Microfluidics separation reveals the stem-cell-like deformability of tumor-initiating cells. Proc. Natl. Acad. Sci. USA.

[212] Ma N., Kamalakshakurup G., Aghaamoo M., Lee A.P., Digman M.A. (2019). Label-Free Metabolic Classification of Single Cells in Droplets Using the Phasor Approach to Fluorescence Lifetime Imaging Microscopy. Cytometry A.

[213] Argüello-Miranda O., Marchand A.J., Kennedy T., Russo M.A.X., Noh J. (2022). Cell cycle–independent integration of stress signals by Xbp1 promotes Non-G1/G0 quiescence entry. J. Cell Biol..

[214] Kang D.-K., Lu J., Zhang W., Chang E., Eckert M.A., Ali M.M., Zhao W., Li X. (2021). Microfluidic devices for stem cell analysis. Microfluidic Devices for Biomedical Applications.

[215] Liu B., Wang X., Jiang L., Xu J., Zohar Y., Yao G. (2021). Extracellular Fluid Flow Induces Shallow Quiescence through Physical and Biochemical Cues. biorXiv.

[216] Fares J.E., El Tomb P., Khalil L.E., Atwani R.W., Moukadem H.A., Awada A., El Saghir N.S. (2020). Metronomic chemotherapy for patients with metastatic breast cancer: Review of effectiveness and potential use during pandemics. Cancer Treat. Rev..

[217] Foldi J., Silber A., Reisenbichler E., Singh K., Fischbach N., Persico J., Adelson K., Katoch A., Horowitz N., Lannin D. (2021). Neoadjuvant durvalumab plus weekly nab-paclitaxel and dose-dense doxorubicin/cyclophosphamide in triple-negative breast cancer. Npj Breast Cancer.

[218] McClendon A.K., Dean J.L., Rivadeneira D.B., Yu J.E., Reed C.A., Gao E., Farber J.L., Force T., Koch W.J., Knudsen E.S. (2012). CDK4/6 inhibition antagonizes the cytotoxic response to anthracycline therapy. Cell Cycle.

[219] Petrelli F., Ghidini A., Pedersini R., Cabiddu M., Borgonovo K., Parati M.C., Ghilardi M., Amoroso V., Berruti A., Barni S. (2019). Comparative efficacy of palbociclib, ribociclib and abemaciclib for ER+ metastatic breast cancer: An adjusted indirect analysis of randomized controlled trials. Breast Cancer Res. Treat..

[220] Shah M., Nunes M.R., Stearns V. (2018). CDK4/6 Inhibitors: Game Changers in the Management of Hormone Receptor–Positive Advanced Breast Cancer?. Oncology.

[221] Zhong L., Li Y., Xiong L., Wang W., Wu M., Yuan T., Yang W., Tian C., Miao Z., Wang T. (2021). Small molecules in targeted cancer therapy: Advances, challenges, and future perspectives. Signal Transduct. Target. Ther..

[222] von Minckwitz G., Huang C.-S., Mano M.S., Loibl S., Mamounas E.P., Untch M., Wolmark N., Rastogi P., Schneeweiss A., Redondo A. (2019). Trastuzumab Emtansine for Residual Invasive HER2-Positive Breast Cancer. N. Engl. J. Med..

[223] Eyvazi S., Farajnia S., Dastmalchi S., Kanipour F., Zarredar H., Bandehpour M. (2018). Antibody Based EpCAM Targeted Therapy of Cancer, Review and Update. Curr. Cancer Drug Targets.

[224] Pondé N., Aftimos P., Piccart M. (2019). Antibody-Drug Conjugates in Breast Cancer: A Comprehensive Review. Curr. Treat. Options Oncol..

[225] Philipson B.I., O’Connor R.S., May M.J., June C.H., Albelda S.M., Milone M.C. (2020). 4-1BB costimulation promotes CAR T cell survival through noncanonical NF-κB signaling. Sci. Signal..

[226] Guedan S., Posey A.D., Shaw C., Wing A., Da T., Patel P.R., McGettigan S.E., Casado-Medrano V., Kawalekar O.U., Uribe-Herranz M. (2018). Enhancing CAR T cell persistence through ICOS and 4-1BB costimulation. JCI Insight.

[227] Zhang H., Li F., Cao J., Wang X., Cheng H., Qi K., Wang G., Xu K., Zheng J., Fu Y.-X. (2021). A chimeric antigen receptor with antigen-independent OX40 signaling mediates potent antitumor activity. Sci. Transl. Med..

[228] Melzer M., Lopez-Martinez A., Altomonte J. (2017). Oncolytic Vesicular Stomatitis Virus as a Viro-Immunotherapy: Defeating Cancer with a “Hammer” and “Anvil. ” Biomedicines.

[229] Pol J.G., Lévesque S., Workenhe S.T., Gujar S., Le Boeuf F., Clements D.R., Fahrner J.-E., Fend L., Bell J.C., Mossman K.L. (2018). Trial Watch: Oncolytic viro-immunotherapy of hematologic and solid tumors. OncoImmunology.

[230] Zamani P., Navashenaq J.G., Nikpoor A.R., Hatamipour M., Oskuee R.K., Badiee A., Jaafari M.R. (2019). MPL nano-liposomal vaccine containing P5 HER2/neu-derived peptide pulsed PADRE as an effective vaccine in a mice TUBO model of breast cancer. J. Controlled Release.

[231] Shang M., Soon R.H., Lim C.T., Khoo B.L., Han J. (2019). Microfluidic modelling of the tumor microenvironment for anti-cancer drug development. Lab. Chip.

[232] Venugopal Menon N., Lim S.B., Lim C.T. (2019). Microfluidics for personalized drug screening of cancer. Curr. Opin. Pharmacol..

[233] Yang Y., Yang X., Zou J., Jia C., Hu Y., Du H., Wang H. (2015). Evaluation of photodynamic therapy efficiency using an in vitro three-dimensional microfluidic breast cancer tissue model. Lab. Chip.

[234] Chen Y., Gao D., Wang Y., Lin S., Jiang Y. (2018). A novel 3D breast-cancer-on-chip platform for therapeutic evaluation of drug delivery systems. Anal. Chim. Acta.

[235] Ozcelikkale A., Moon H., Linnes M., Han B. (2017). In vitro microfluidic models of tumor microenvironment to screen transport of drugs and nanoparticles: In vitro microfluidic models of tumor microenvironment. Wiley Interdiscip. Rev. Nanomed. Nanobiotechnol..

[236] Tomeh M.A., Zhao X. (2020). Recent Advances in Microfluidics for the Preparation of Drug and Gene Delivery Systems. Mol. Pharm..

[237] Sharma I., Thakur M., Singh S., Tripathi A. (2021). Microfluidic Devices as a Tool for Drug Delivery and Diagnosis: A Review. Int. J. Appl. Pharm..

[238] Fadhil Majnis M., Francis H., Zilati Ku Shaari K. (2018). Droplet formation in microchannels at low values of the capillary and the reynolds numbers. Mater. Today Proc..

[239] He J., Wei Z., Wang L., Tomova Z., Babu T., Wang C., Han X., Fourkas J.T., Nie Z. (2013). Hydrodynamically Driven Self-Assembly of Giant Vesicles of Metal Nanoparticles for Remote-Controlled Release. Angew. Chem. Int. Ed..

[240] Lou G., Anderluzzi G., Woods S., Roberts C.W., Perrie Y. (2019). A novel microfluidic-based approach to formulate size-tuneable large unilamellar cationic liposomes: Formulation, cellular uptake and biodistribution investigations. Eur. J. Pharm. Biopharm..

[241] Liu K., Zhu Z., Wang X., Gonçalves D., Zhang B., Hierlemann A., Hunziker P. (2015). Microfluidics-based single-step preparation of injection-ready polymeric nanosystems for medical imaging and drug delivery. Nanoscale.

[242] Liu D., Bernuz C.R., Fan J., Li W., Correia A., Hirvonen J., Santos H.A. (2017). A Nano-in-Nano Vector: Merging the Best of Polymeric Nanoparticles and Drug Nanocrystals. Adv. Funct. Mater..

[243] Li W., Liu D., Zhang H., Correia A., Mäkilä E., Salonen J., Hirvonen J., Santos H.A. (2017). Microfluidic assembly of a nano-in-micro dual drug delivery platform composed of halloysite nanotubes and a pH-responsive polymer for colon cancer therapy. Acta Biomater..

[244] Hajba L., Guttman A. (2017). Continuous-flow-based microfluidic systems for therapeutic monoclonal antibody production and organ-on-a-chip drug testing. J. Flow Chem..

[245] Zhang W., Li Q., Jia F., Hu Z., Wei Z. (2021). A Microfluidic Chip for Screening and Sequencing of Monoclonal Antibody at a Single-Cell Level. Anal. Chem..

[246] Silva D.F.C., Azevedo A.M., Fernandes P., Chu V., Conde J.P., Aires-Barros M.R. (2012). Design of a microfluidic platform for monoclonal antibody extraction using an aqueous two-phase system. J. Chromatogr. A.

[247] Bourguignon N., Attallah C., Karp P., Booth R., Peñaherrera A., Payés C., Oggero M., Pérez M.S., Helguera G., Lerner B. (2018). Production of monoclonal antibodies in microfluidic devices. Integr. Biol..

[248] Wright B.D., Whittenberg J., Desai A., DiFelice C., Kenis P.J.A., Lapi S.E., Reichert D.E. (2016). Microfluidic Preparation of a ^89^ Zr-Labeled Trastuzumab Single-Patient Dose. J. Nucl. Med..

[249] Terrell-Hall T.B., Nounou M.I., El-Amrawy F., Griffith J.I.G., Lockman P.R. (2017). Trastuzumab distribution in an *in-vivo* and *in-vitro* model of brain metastases of breast cancer. Oncotarget.

[250] Wang Y., Jin R., Shen B., Li N., Zhou H., Wang W., Zhao Y., Huang M., Fang P., Wang S. (2021). High-throughput functional screening for next-generation cancer immunotherapy using droplet-based microfluidics. Sci. Adv..

[251] Elsemary M.T., Maritz M.F., Smith L.E., Thierry B. (2019). Microfluidic purification of T lymphocytes separated from blood for chimeric antigen receptor T-cell manufacturing. Cytotherapy.

[252] Fajrial A.K., He Q.Q., Wirusanti N.I., Slansky J.E., Ding X. (2020). A review of emerging physical transfection methods for CRISPR/Cas9-mediated gene editing. Theranostics.

[253] Ide H., Espulgar W.V., Saito M., Aoshi T., Koyama S., Takamatsu H., Tamiya E. (2021). Profiling T cell interaction and activation through microfluidics-assisted serial encounter with APCs. Sens. Actuators B Chem..

[254] Jimeno A., Baranda J., Mita M., Gordon M., Taylor M., Iams W., Janku F., Matulonis U., Bernstein H., Loughhead S. (2021). Initial results of a first-in-human, dose escalation study of a cell-based vaccine in HLA A*02+ patients (pts) with recurrent, locally advanced or metastatic HPV16+ solid tumors: SQZ-PBMC-HPV-101. J. Clin. Oncol..

[255] Booty M., Stockmann A., Pryor O., Myint M., Trumpfheller C., Nicolini V., Klein C., Codarri L., Umana P., Sharei A. (2020). 141 PBMC-based cancer vaccines generated with microfluidics squeezing demonstrate synergistic and durable tumor reduction in combination with PD1 checkpoint and FAP targeted IL-2 variants. J. Immunother. Cancer.

[256] Chao B.H., Sung K.E., Yin J., Beebe D.J. (2011). Abstract C236: The inhibitory effect of tumor microenvironment on oncolytic virotherapy in lung cancer. Mol. Cancer Ther..

[257] Lee S.W., Lee K.J., Jeong S.Y., Joo C.H., Lee H., Jeong G.S. (2020). Evaluation of Bystander Infection of Oncolytic Virus using a Medium Flow Integrated 3D In Vitro Microphysiological System. Adv. Biosyst..

[258] Qiao H., Chen X., Wang Q., Zhang J., Huang D., Chen E., Qian H., Zhong Y., Tang Q., Chen W. (2020). Tumor localization of oncolytic adenovirus assisted by pH-degradable microgels with JQ1-mediated boosting replication and PD-L1 suppression for enhanced cancer therapy. Biomater. Sci..

[259] Paterson K., Zanivan S., Glasspool R., Coffelt S.B., Zagnoni M. (2021). Microfluidic technologies for immunotherapy studies on solid tumours. Lab. Chip.

[260] Lee K.J., Lee S.W., Woo H.-N., Cho H.M., Yu D.B., Jeong S.Y., Joo C.H., Jeong G.S., Lee H. (2020). Real-time monitoring of oncolytic VSV properties in a novel in vitro microphysiological system containing 3D multicellular tumor spheroids. PLoS ONE.

[261] Fan Y., Li M., Ma K., Hu Y., Jing J., Shi Y., Li E., Dong D. (2019). Dual-target MDM2/MDMX inhibitor increases the sensitization of doxorubicin and inhibits migration and invasion abilities of triple-negative breast cancer cells through activation of TAB1/TAK1/p38 MAPK pathway. Cancer Biol. Ther..

[262] Lovitt C.J., Shelper T.B., Avery V.M. (2018). Doxorubicin resistance in breast cancer cells is mediated by extracellular matrix proteins. BMC Cancer.

[263] Jia L., Jia N., Gao Y., Hu H., Zhao X., Chen D., Qiao M. (2019). Multi-Modulation of Doxorubicin Resistance in Breast Cancer Cells by Poly(l-histidine)-Based Multifunctional Micelles. Pharmaceutics.

[264] Marinello P.C., Panis C., Silva T.N.X., Binato R., Abdelhay E., Rodrigues J.A., Mencalha A.L., Lopes N.M.D., Luiz R.C., Cecchini R. (2019). Metformin prevention of doxorubicin resistance in MCF-7 and MDA-MB-231 involves oxidative stress generation and modulation of cell adaptation genes. Sci. Rep..

[265] Chen Y., Cai H., Chen W., Guan Q., He J., Guo Z., Li J. (2020). A Qualitative Transcriptional Signature for Predicting Extreme Resistance of ER-Negative Breast Cancer to Paclitaxel, Doxorubicin, and Cyclophosphamide Neoadjuvant Chemotherapy. Front. Mol. Biosci..

[266] Merry C.R., McMahon S., Forrest M.E., Bartels C.F., Saiakhova A., Bartel C.A., Scacheri P.C., Thompson C.L., Jackson M.W., Harris L.N. (2016). Transcriptome-wide identification of mRNAs and lincRNAs associated with trastuzumab-resistance in HER2-positive breast cancer. Oncotarget.

[267] Yazdanifar M., Zhou R., Grover P., Williams C., Bose M., Moore L., Wu S., Maher J., Dreau D., Mukherjee P. (2019). Overcoming Immunological Resistance Enhances the Efficacy of A Novel Anti-tMUC1-CAR T Cell Treatment against Pancreatic Ductal Adenocarcinoma. Cells.

[268] Chaurasiya S., Fong Y. (2021). Viroimmunotherapy for breast cancer: Promises, problems and future directions. Cancer Gene Ther..

[269] Shah N.N., Fry T.J. (2019). Mechanisms of resistance to CAR T cell therapy. Nat. Rev. Clin. Oncol..

[270] Garg A.D., Elsen S., Krysko D.V., Vandenabeele P., de Witte P., Agostinis P. (2015). Resistance to anticancer vaccination effect is controlled by a cancer cell-autonomous phenotype that disrupts immunogenic phagocytic removal. Oncotarget.

[271] Liu D., Wang L., Zhong R., Li B., Ye N., Liu X., Lin B. (2007). Parallel microfluidic networks for studying cellular response to chemical modulation. J. Biotechnol..

[272] Sarkar S., Cohen N., Sabhachandani P., Konry T. (2015). Phenotypic drug profiling in droplet microfluidics for better targeting of drug-resistant tumors. Lab. Chip.

[273] Ozcelikkale A., Shin K., Noe-Kim V., Elzey B.D., Dong Z., Zhang J.-T., Kim K., Kwon I.C., Park K., Han B. (2017). Differential response to doxorubicin in breast cancer subtypes simulated by a microfluidic tumor model. J. Controlled Release.

[274] Qi R., Zhu G., Wang Y., Wu S., Li S., Zhang D., Bu Y., Bhave G., Han R., Liu X. (2019). Microfluidic device for the analysis of MDR cancerous cell-derived exosomes’ response to nanotherapy. Biomed. Microdevices.

[275] Parekh K., Noghabi H.S., Lopez J.A., Li P.C.H. (2020). Microfluidic chip enables single-cell measurement for multidrug resistance in triple-negative breast cancer cells. Cancer Drug Resist.

[276] Parekh K., Sharifi H., Khamenehfar A., Beischlag T.V., Payer R.T.M., Li P.C.H. (2019). The microfluidic capture of single breast cancer cells for multi-drug resistance assays. Methods in Enzymology.

[277] Rahman S.M., Campbell J.M., Coates R.N., Render K.M., Byrne C.E., Martin E.C., Melvin A.T. (2020). Evaluation of intercellular communication between breast cancer cells and adipose-derived stem cells *via* passive diffusion in a two-layer microfluidic device. Lab. Chip.

